# Bacillus Calmette–Guérin (BCG) immunotherapy reprograms CNS immunity and alters Alzheimer’s biomarkers: results from two open-label clinical trials

**DOI:** 10.1038/s43856-026-01691-7

**Published:** 2026-07-02

**Authors:** Marc S. Weinberg, Mahesh Chandra Kodali, Zhaozhi Li, Jake A. Galler, Arianna R. Tidball, William Cody Reynolds, Cathrine Young, Kelli Devitte-McKee, Hadia A. Fatima, Pia Kivisäkk, Mandovi Chatterjee, Arpita Kulkarni, James M. Billingsly, Alexandra L. Bartlett, Shannan Ho Sui, Willem M. Kühtreiber, Jessica Gerber, Alison J. McManus, Rudolph E. Tanzi, Sudeshna Das, Denise L. Faustman, Steven E. Arnold

**Affiliations:** 1 Massachusetts General Hospital, Boston, MA USA; 2 Harvard Medical School, Boston, MA USA; 3 Department of Computational Biology, Cornell University, Ithaca, NY USA; 4 Harvard T.H. Chan School of Public Health, Boston, MA USA; 5 Department of Neurology, Genetics and Aging Research Unit, McCance Center for Brain Health, Massachusetts General Hospital, Boston, MA USA

**Keywords:** Alzheimer's disease, Live attenuated vaccines

## Abstract

**Background:**

Immune aging may contribute to Alzheimer’s disease. Bacillus Calmette–Guérin (BCG), a vaccine known to induce trained immunity, has been linked to reduced Alzheimer’s risk in prior studies. However, whether trained immunity can be observed in the human central nervous system remains unclear. We assessed whether BCG induces trained immunity–like responses in adults with and without Alzheimer’s-related changes.

**Methods:**

We conducted two related one-year, open-label clinical trials in adults aged 55 years or older (*n* = 12 without Alzheimer’s-related pathology; *n* = 11 with Alzheimer’s-related pathology) recruited at a single center. Participants received two intradermal BCG vaccinations one month apart. Protocol-defined objectives included safety, neurocognitive outcomes, and longitudinal immune and Alzheimer’s biomarker changes in blood and cerebrospinal fluid. Immune responses were assessed using cytokine assays and single-cell profiling. All enrolled participants were included where data were available; longitudinal changes were analyzed using mixed-effects models.

**Results:**

Here we show that BCG induces persistent, trained immunity–like changes in immune cells in cerebrospinal fluid, including enhanced innate responsiveness and associated transcriptional programs. These responses differ from blood, suggesting compartment-specific immune imprinting. In participants without Alzheimer’s-related changes, these immune shifts are accompanied by decreased amyloid-β levels in cerebrospinal fluid and increased levels in blood. BCG was well tolerated, with no unexpected safety signals observed.

**Conclusions:**

These findings suggest trained immunity–like responses in the central nervous system that may influence Alzheimer’s-relevant pathways. This approach may represent an early neurodegenerative intervention strategy, although larger controlled studies are needed to confirm these observations.

**Trial registration:**

ClinicalTrials.gov NCT04507126 (June 23, 2020) and NCT05004688 (August 6, 2021).

## Plain language summary

As people age, the immune system becomes less effective and may contribute to diseases like Alzheimer’s disease, a condition that affects memory and thinking. The BCG vaccine, commonly used against tuberculosis, can “train” the immune system to respond better to challenges. In this study, we tested whether BCG could change immune activity in both the blood and the fluid surrounding the brain and spinal cord in older adults with and without Alzheimer’s-related changes. Participants received BCG and were followed for one year with blood and spinal fluid tests. We found that BCG changed how immune cells behaved, including in the fluid surrounding the brain and spinal cord, and altered markers linked to Alzheimer’s disease. These findings suggest that boosting the immune system in specific ways may help maintain brain health during aging, but larger studies are needed to understand whether this could prevent or treat disease.

## Abstract (web summary)

Weinberg, Kodali, Li et al. investigate the effects of BCG (tuberculosis) vaccination on brain and blood immune responses and Alzheimer’s-related biomarkers in older adults. They find durable immune reprogramming (“trained immunity”) in CSF cells and associated changes in amyloid-β dynamics.

## Introduction

Aging is marked by immune remodeling that alters disease resilience. A key feature is “inflammaging”, a state of chronic, low-grade inflammation linked to impaired tissue repair and neurodegeneration^1–3^. Alzheimer’s disease (AD) exemplifies this process, with impaired amyloid-β (Aβ) clearance, sustained neuroinflammation, and systemic immune dysregulation contributing to disease progression^4,5^. In the central nervous system (CNS), microglia, perivascular macrophages, and infiltrating monocytes undergo age-related metabolic shifts that impair immune function^6–8^. Mitochondrial dysfunction further disrupts immune function, amplifying inflammation in aging and AD^9,10^.

Trained immunity—innate immune memory driven by metabolic and epigenetic reprogramming—offers a potential strategy to enhance immune resilience in aging and neurodegeneration^11^. It was first recognized after Bacillus Calmette–Guérin (BCG) vaccination, where protection against unrelated infections emerged in clinical and epidemiological studies. These nonspecific effects were later attributed to long-term functional changes in innate immune cells^12^. BCG is now a well-characterized inducer of trained immunity, known to boost antimicrobial defenses and promote long-term immunoregulation^13–17^. Retrospective studies report reduced AD incidence among bladder cancer patients treated with BCG^18–21^. In preclinical models, BCG reduces Aβ pathology, suppresses neuroinflammation, and preserves synaptic integrity^22–25^. Mechanistically, BCG reprograms monocytes toward aerobic glycolysis, enhancing cytokine production and recall responses^26,27^. Recent evidence also suggests longer-term transcriptional and metabolic effects in lymphoid cells, including CD4^+^ T cells^28,29^. Together, these findings suggest BCG immunotherapy may counteract immune dysfunction and modify pathways relevant to AD pathophysiology.

Despite these promising findings, the impact of BCG-induced trained immunity on CNS-resident and CNS-associated immune populations—and its relevance to AD pathophysiology—remains unclear. While BCG reprograms peripheral monocytes and other innate immune subsets, its effects on neuroinflammation and AD biomarkers in humans remain largely unexplored. We hypothesized that BCG immunotherapy would induce trained immunity in both peripheral and CNS immune compartments, leading to modulation of inflammatory mediators and Alzheimer’s-related biomarkers. To test this, we conducted a one-year, open-label study of BCG immunotherapy in older adults with and without AD pathology. We assessed trained immunity signatures in peripheral and central compartments (via cerebrospinal fluid (CSF) profiling), including modulation of inflammatory cytokines, AD-related biomarkers, and transcriptional remodeling over 12 months.

We find that BCG is associated with sustained immune remodeling in older adults, including trained immunity signatures in peripheral and CSF compartments, modulation of inflammatory cytokines, and changes in Alzheimer’s-related biomarkers. BCG induces persistent transcriptional shifts in CNS–associated immune cells, including enrichment of immune and metabolic pathways. These findings provide evidence of trained immunity–like responses in the CNS in humans and suggest that immune reprogramming may represent a strategy to target immune dysfunction and early neurodegeneration.

## Methods

### Study design

Two open-label clinical trials were conducted under FDA IND 23605, each approximately one year in duration and distinguished by study population. The trials were registered on ClinicalTrials.gov (NCT04507126, registered June 23, 2020; https://clinicaltrials.gov/study/NCT04507126; NCT05004688, registered August 6, 2021; https://clinicaltrials.gov/study/NCT05004688), and governed by three sequential IRB protocols approved by the Mass General Brigham IRB (2020P002042, 2021P001980, and 2021P002577). These IRB protocols reflected the original design and a subsequent extension of NCT04507126, as well as the separate initiation of NCT05004688.

The first trial (NCT04507126) enrolled cognitively unimpaired and mildly impaired older adults. It began as a 3-month observational study and was later extended to a total 12-month follow-up. The second trial (NCT05004688) enrolled individuals with mild cognitive impairment (MCI) or mild-to-moderate Alzheimer’s disease (AD). This MCI/AD trial was originally designed as a randomized study but was conducted as a single-arm, open-label trial due to feasibility and funding constraints. All procedures and informed consent materials were approved by the Mass General Brigham IRB prior to enrollment, and all participants, or their legally authorized representatives when applicable, provided written informed consent prior to study participation. Patients and the public were not involved in the design, conduct, reporting, or dissemination plans of this study. The study was conducted at a single academic medical center (Massachusetts General Hospital, Boston, MA, USA) in an outpatient research setting. No formal sample size calculation was performed. All study procedures, including BCG administration, were performed by trained clinical research personnel according to institutional protocols.

#### Eligibility criteria

Eligibility criteria differed between the two studies. In NCT04507126, participants were aged 55–80 with MoCA scores ≥18 and no more than mild cognitive symptoms. In NCT05004688, participants were aged 55–85 and met criteria for mild cognitive impairment or mild-to-moderate Alzheimer’s disease, including MoCA ≥8, a Clinical Dementia Rating (CDR) global score between 0.5 and 2, a study partner available for all visits, and biomarker-confirmed AD pathology (via amyloid PET, CSF Aβ42/40 ratio, or confirmed by CSF immunoassay during screening).

Across both studies, participants were required to be medically stable, able to provide informed consent and undergo study procedures including lumbar puncture, and free of conditions or treatments associated with increased infection risk. Key exclusions included prior BCG vaccination or tuberculosis exposure, immunosuppression or immunomodulatory therapies, recent metformin use (within 12 months), given its potential interaction with BCG-induced metabolic reprogramming^30^, active or chronic infectious disease, clinically significant neurological or psychiatric comorbidity, and laboratory safety criteria (including hematologic, metabolic, and coagulation parameters), infection screening (e.g., tuberculosis and HIV), and clinical findings that would increase risk from vaccination or study procedures. Additional protocol-defined criteria, including detailed laboratory thresholds, medication restrictions, and reproductive considerations, are provided in the study protocols (See Supplementary Information for complete protocols).

#### Participation timelines

Participation in NCT04507126 and NCT05004688 spanned from December 2020 to December 2022 and from April 2022 to January 2024, respectively. These timelines reflect the full duration from initial screening through completion of study visits and biospecimen collection; downstream laboratory analyses (e.g., cytokine profiling, PBMC stimulation assays, single-cell RNA sequencing) were performed thereafter.

### Intervention and assessment schedule

**Fig. 1:**
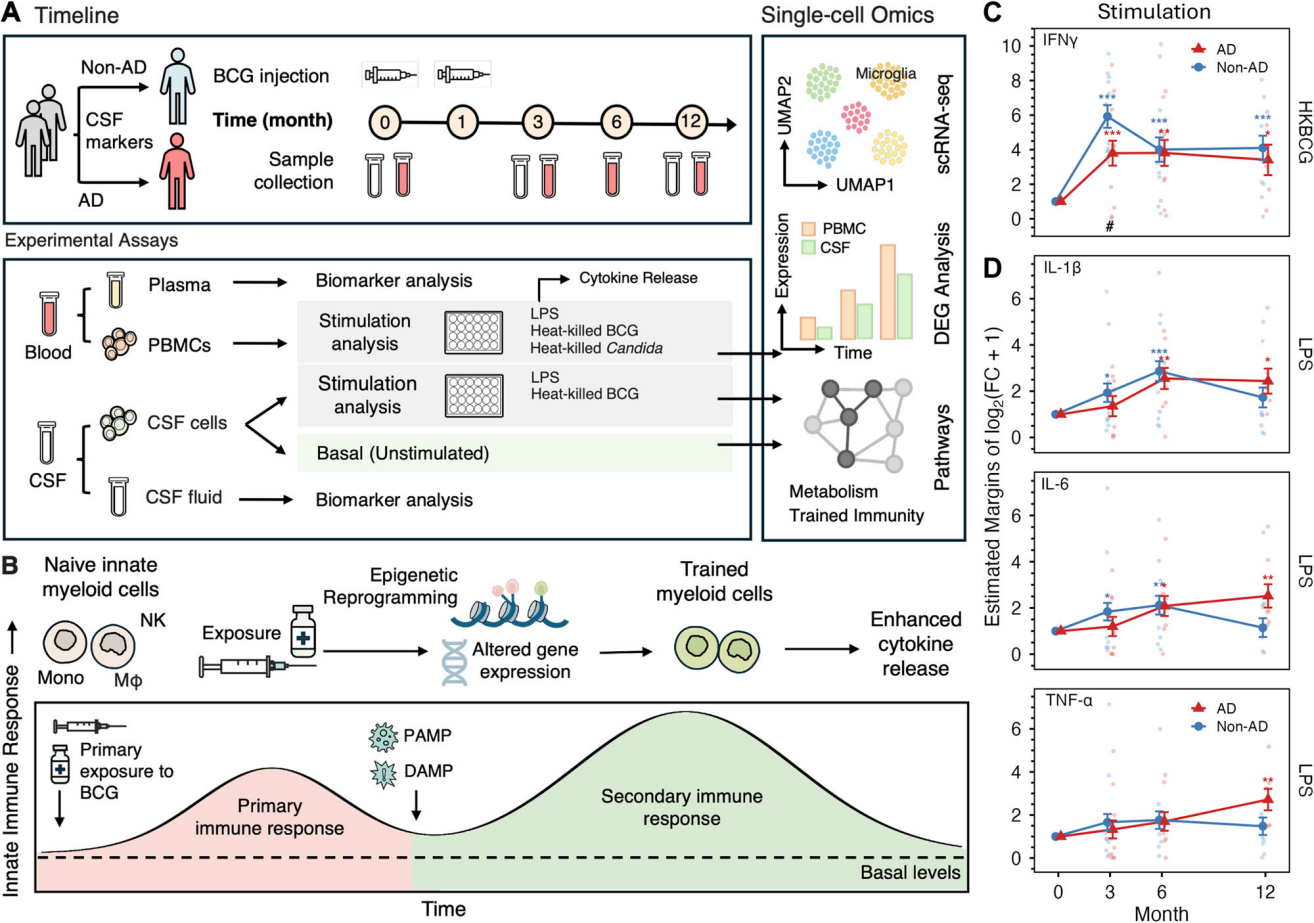
Study design and trained immunity-associated cytokine responses in PBMCs. **A** Study design. Older adults with and without AD pathology received intradermal BCG at baseline and one month. Blood and cerebrospinal fluid (CSF) were collected at baseline, 3 months, 6 months (blood only), and 12 months. Peripheral blood mononuclear cells (PBMCs) and CSF monocytes were isolated for stimulation assays with lipopolysaccharide (LPS), heat-killed BCG (HKBCG), and heat-killed *Candida albicans* (HKCA, PBMC only). Cytokines and biomarkers were measured in plasma, CSF, and PBMC stimulation supernatants. Select basal and stimulated CSF and PBMC cells were profiled by single-cell omics. **B** Model of trained immunity: innate immune training via BCG leads to epigenetic reprogramming and increased responsiveness to subsequent pathogen-associated molecular patterns (PAMPs) or damage-associated molecular patterns (DAMPs). **C** HKBCG stimulation of PBMCs increased IFN-γ at all post-baseline timepoints, with early peaks in non-AD and sustained elevations in AD participants. **D** LPS stimulation significantly increased IL-1β, IL-6, and TNF-α in both groups, with responses sustained through 12 months only in AD participants. Data represent estimated marginal means of log₂(fold change +1) from linear mixed-effects models with fixed effects for Month, AD status, and their interaction, and subject-level random intercepts. Asterisks indicate within-group significance compared to baseline (^*^p < 0.05, ^**^p < 0.01, ^***^p < 0.001); hash marks indicate AD × Month interaction (^#^*p* < 0.05, ^##^*p* < 0.01, ^###^*p* < 0.001). Error bars show standard error of the mean (SEM). A lower baseline response to HKBCG was observed in AD vs. non-AD participants for IFN-γ (Supplementary Data 3); no baseline group differences were observed for LPS responses. Exact annotated *p* values for within-group comparisons versus baseline were: IFN-γ non-AD (3m *p* < 0.001, 6m *p* < 0.001, 12m *p* < 0.001), AD (3m *p* < 0.001, 6m *p* = 0.001, 12m *p* = 0.011); IL-1β non-AD (3m *p* = 0.041, 6m *p* < 0.001), AD (6m *p* = 0.003, 12m *p* = 0.016); IL-6 non-AD (3m *p* = 0.038, 6m *p* = 0.009), AD (6m *p* = 0.018, 12m *p* = 0.004); TNF-α AD (12m *p* = 0.001). The exact annotated AD × Month interaction *p* value at 3m for IFN-γ was 0.049. Sample sizes vary by analyte and timepoint; full model Ns, effect estimates, 95% confidence intervals, and exact *p* values are reported in Supplementary Data 4. All statistical tests were two-sided; *p* values are nominal and not adjusted for multiple comparisons, consistent with the exploratory design.

Participants received two intradermal vaccinations at baseline and one month later. Phlebotomy and lumbar puncture were performed at baseline, 3 months, and 12 months. An additional blood draw at 6 months was conducted without a lumbar puncture (Fig. 1A). Adverse events were prospectively collected throughout the study. Cognitive and neuropsychological testing (MoCA, CDR, RBANS, FAQ, NPI-Q) was performed longitudinally.

#### Adverse event collection and classification

Adverse events were monitored continuously from the time of informed consent through study completion. At each visit, participants were queried for interval symptoms or medical events, and findings were recorded in case report forms. Adverse events included clinical events and clinically significant laboratory abnormalities and were classified by investigators according to prespecified relatedness categories (not related, unlikely, possibly, probably, or definitely related).

#### BCG treatment and monitoring

GMP-quality Tokyo 172 strain of Mycobacterium bovis BCG (Japan BCG Laboratory (JBL), Tokyo, Japan, 1.8–3.9 × 10^6^ colony forming units/injection) was reconstituted in 0.9% sodium chloride for a total volume 0.1 mL. Intradermal injections were given to the right (baseline) and left (1 month) deltoid.

### Biospecimen collection and processing

#### Phlebotomy and peripheral blood mononuclear cell (PBMC) isolation

Whole blood was collected into EDTA-coated, purple-colored vacutainer tubes (BD, Cat# 366643). PBMCs were isolated from the whole blood using density gradient centrifugation. Whole blood was centrifuged at 1500 × *g* at room temperature for 10 min to separate plasma. Plasma was then frozen in 0.5 mL aliquots at −80 °C. The resulting blood pellet was then reconstituted to the original volume and diluted 2× with pre-warmed wash buffer (phosphate-buffered saline supplemented with 2% fetal bovine serum (Gibco, Cat# A4766801)). Fractions of 30 mL of the diluted blood were layered atop 15 mL Lymphoprep density gradient medium (Stem cell, Cat# 07801) in a 50 mL Falcon tube and centrifuged at 800 × *g* without brake at room temperature for 30 min. Cells were carefully harvested from the interface and then washed 2× with the wash buffer before resuspending into 10 mL wash buffer. Cells were then counted using Luna-FL automated cell counter with acridine orange and propidium iodide viability dye (Logos bio, Cat# F23001). Cells were cryopreserved at 1 × 10^7^ cells/mL in FBS + 10% DMSO.

#### Lumbar puncture and CSF processing

Lumbar punctures (LPs) were performed after no less than 8 h fasting. 1% lidocaine was used as local anesthetic, and up to 25 mL CSF was collected by drip into a polystyrene tube using a 22G Whitacre atraumatic needle. To avoid red blood cell contamination, the first 1–3 mL of CSF was discarded until visibly clear fluid was obtained. CSF was collected in ice-chilled 15 mL Falcon tubes and centrifuged within 30 min of collection. Samples were centrifuged at 3000 × *g* for 10 min at 4 °C using a swinging bucket rotor. All but 0.1 mL of fluid was aspirated into another tube (acellular CSF, for fluid-based biomarker studies), and the cell pellet was resuspended. Acellular CSF was immediately aliquoted into 0.5 mL aliquots and frozen at −80 °C. Cell viability and cell count were performed on pelleted cells using Luna-FL automated cell counter with acridine orange and propidium iodide viability dye (Logos bio, Cat# F23001). Red blood cell contamination was estimated by subtracting nucleated from total cells. Cells were treated with 1× DNase (Stem cell, Cat# 07900) for 30 min at 22 °C, then washed and spun at 3000 × *g* for 6 min, followed by cryopreservation in fetal bovine serum (FBS, Thermo-scientific, Cat# A4766801) mixed with 10% DMSO (Sigma Aldrich, Cat# D2650). Our previous work demonstrated the preservation of transcriptomic integrity using FBS/DMSO cryopreservation of CSF cells^31^.

### Stimulation assays and immunoassays

#### PBMC stimulation assay

Cryopreserved cells were warmed to near complete thaw in a 37 °C water bath, after which 1.5 mL pre-warmed X-VIVO 15 media (Lonza, cat #418Q) with Gentamicin and Phenol Red (hereafter referred to as *media*) was added to the cryovial. Cells were resuspended and transferred to a 15 mL falcon tube containing 9 mL pre-warmed media. Falcon tubes were then centrifuged at 300 × *g* at room temperature for 8 min, and the supernatant was removed. Cells were then washed twice with 12 mL of media to remove any traces of DMSO. Finally, cells were resuspended in 3 mL of media and transferred to a new 15 ml falcon tube. 30 μL of the cell suspension was removed and analyzed for viability and cell count using Luna-FL automated cell counter with acridine orange and propidium iodide viability dye (Logos bio, cat #F23001). Cells were then rested overnight for recovery in media at 37 °C in a 5% CO_2_ incubator. Following the overnight recovery, cells were again washed 2× with pre-warmed media and counted. 200,000 cells were plated into each well in a 96-well and treated for 6 h with LPS (1 ng/mL), heat-killed *Candida albicans* (HKCA, 1e5 cfu/mL), and for 24 h with heat-killed BCG (HKBCG, 1e4 cfu/mL). Following the stimulation at 37 °C in a 5% CO_2_ incubator, cell-culture supernatant was harvested and stored at −80 °C for cytokine analysis.

#### Immunoassays

*PBMC media cytokine analysis*: Levels of IL-1β, IL-6, TNF-α, and IFN-γ in media of stimulated PBMCs were measured using MSD V-PLEX (Meso-Scale Discovery (MSD), cat K151A9H, K151QOD) following the manufacturer’s instructions, using a 4× dilution. *Plasma and CSF cytokine analysis:* Plasma and CSF cytokine analysis were performed using MSD S-PLEX (MSD, K151AKV-2) to detect IFN-γ, IL-1β, IL-2, IL-6, IL-10, and TNF-α analytes, following the manufacturer’s instructions, without any dilution. *APOEε4 immunoassay*: APOEε4 allelic frequency estimation was estimated from plasma using a Human Apolipoprotein E4 ELISA Kit (Abcam, Cat# ab285256), by following the manufacturer’s protocol (plasma diluted 100×, APOEε4 positive controls included for relative comparison). *AD Biomarkers:* CSF samples from individuals requiring biomarker evidence of AD as related to inclusion criteria were submitted for Fujirebio Lumipulse Amyloid-β 42 and 40 immunoassay. A research threshold of ≤0.067 defined positive AD pathology solely for inclusion criteria, as established through a sliding window algorithm, using a cohort of AD-diagnosed subjects and cognitively unimpaired individuals from the Massachusetts General Hospital (MGH) MIND clinic, having 92% sensitivity and specificity^32^. Quantitative analyses of CSF and Plasma AD biomarkers were completed using Quanterix Simoa® HD-X (kits 104465, 101995, 104111). All longitudinal samples from a given participant (baseline and follow-up timepoints) were assayed on the same plate to minimize inter-assay variability. *Data normalization*: Bridge samples were included in all immunoassays, and batch normalization was applied to all results through linear regression. Because all longitudinal samples from a given participant were run on the same plate, batch effects do not confound within-subject longitudinal comparisons. The post-normalization analyte coefficient of variation (CV) for CSF IL-1β was unexpectedly high (36.7%). All other CVs were <20%. Average CVs for included AD biomarkers and cytokines were 7.9% (CSF) and 8.5% (Plasma).

### Statistical analysis

#### Statistics and reproducibility

This was an exploratory, open-label study across two IRB-approved protocols. The statistical analysis plan (SAP) was embedded within the approved protocols, and no separate standalone SAP was created. No prespecified hypothesis testing or power calculations were planned. All analyses should therefore be considered exploratory unless explicitly specified as prespecified within the original protocol. Sample size was determined by feasibility and reflects the exploratory nature of the study, with the aim of generating immunological and other disease-related biomarker data. No interim analyses or formal stopping guidelines were prespecified or implemented.

Mixed-effects modeling for the MCI/AD protocol was prespecified, whereas application of MMRM to the initial protocol and pooled analyses across protocols were conducted post hoc to harmonize longitudinal analyses. For the MCI/AD protocol (NCT05004688), a mixed-effects model for repeated measures (MMRM) was prespecified and implemented. For the initial cognitively unimpaired/mild cognitive impairment study (NCT04507126), which originally described only paired *t*-tests or Wilcoxon signed-rank tests, MMRM was later applied post hoc to harmonize analysis across timepoints and to enable stratification by AD biomarker status. These adaptations remained consistent with the protocol’s exploratory intent and did not alter study conduct. Analyses were conducted on the pooled cohort to enable consistent longitudinal modeling across timepoints. To assess robustness to protocol differences, sensitivity analyses including protocol as a covariate were performed and did not materially alter the magnitude, direction, or significance of key findings.

#### Data transformation and handling

Immunoassay values were ln-transformed for normalization. Since raw Aβ-related analyses were consistent with log-transformed results, raw values are reported for clinical interpretability. Cytokine stimulation assays were analyzed using a log₂(fold change + 1) transformation to stabilize variance. Single-cell immune cell type proportions were logit-transformed to meet model assumptions, though raw proportions are shown in figures. No available data were excluded. Sample size discrepancies reflect lumbar puncture failures or rare biomaterial or assay loss (see Supplementary Fig. 1 for participant flow and overall sample availability; Supplementary Data 1 for sample-level missingness).

#### Group classification and AD designation

Alzheimer’s disease (AD) designation in this study reflects a biomarker-based classification and does not represent a formal clinical diagnosis. K-means clustering (*k* = 2) was applied to baseline CSF pTau181/Aβ42 ratios using scikit-learn (version 1.6.0^33^) in Python (random_state = 42). Cluster labels were ordered by centroid values to define higher and lower groups. This ratio showed stronger alignment with MoCA-based classifications than CSF Aβ42/40 (Adjusted Rand Index: 0.525 vs. 0.288; Normalized Mutual Information: 0.450 vs. 0.246). Of individuals designated as AD by biomarkers, 90.91% had MoCA scores <26; among non-AD participants, 83.33% had scores ≥26. A comparison of biomarker- and MoCA-based classifications is shown in Supplementary Fig. 2.

#### Baseline comparisons

Due to widespread non-normality (assessed by the Shapiro–Wilk test^34^), two-sided Wilcoxon rank-sum tests were used for all baseline group comparisons. Median and interquartile range values are reported, along with rank-sum z-statistics and exact *p*-values. Effect sizes were calculated using the rank biserial correlation (*r* = *z*/√*N*, where *N* is the total sample size^35^). All baseline data reflect independent samples collected at Month 0. Baseline demographic and clinical characteristics are additionally presented in Supplementary Data 2, stratified by protocol to reflect differences in cohort composition across studies. Results of baseline statistical comparisons are provided in Supplementary Data 3. Standardized mean differences were additionally calculated to assess baseline group balance.

#### Longitudinal mixed-effects modeling

Longitudinal analyses were conducted using linear mixed-effects models fit with the mixed command in StataBE 18.0^36^. Two models were tested for each outcome: (1) a Time-only model, with timepoint as a categorical fixed effect; and (2) an AD × Time model, including fixed effects for AD status, time, and their interaction. The interaction model was retained when significant at any time point (*p* < 0.05); otherwise, the Time-only model was reported. All graphical displays and marginal estimates were stratified by AD status for consistency.

Subject-level random intercepts were included to account for repeated measures. Covariates (sex, age, APOEε4 status) were evaluated via sequential likelihood ratio tests but were excluded due to lack of consistent effect across models. To assess robustness to study-level differences, longitudinal mixed-effects models were repeated with protocol included as a fixed effect in post hoc sensitivity analyses. Models assume missing at random (MAR); all available observations were included without imputation. Model assumptions were evaluated by inspection of residuals. Post-estimation marginal means and standard errors were computed using the margins command. Within-group changes were assessed via the contrast command, and interaction effects were derived from model-fixed coefficients. Models were fit using restricted maximum likelihood. Full model outputs, including contrasts, are reported in Supplementary Data 4, and protocol sensitivity analyses are reported in Supplementary Data 5.

#### CSF-to-plasma Aβ42 ratio analysis

To characterize relative changes in Aβ42 across compartments, we calculated the CSF/plasma Aβ42 ratio at baseline, 3 months, and 12 months for participants with paired samples. The ratio was defined as CSF Aβ42 divided by plasma Aβ42 (using raw values without transformation). Changes in this ratio were analyzed using a linear mixed-effects model with fixed effects for AD status, time (categorical), and their interaction, and random intercepts for subjects. Marginal means from the model were used to compute percent change from baseline. This ratio was interpreted descriptively to evaluate relative Aβ dynamics across compartments and was not treated as a direct marker of clearance or specific mechanistic processes.

#### Interpretation and sensitivity considerations

Figures display data stratified by AD and non-AD groups for transparency. All comparisons are shown visually, though models are reported based on statistical criteria. Given the small sample size and exploratory nature of the study, covariates were excluded for parsimony, and unadjusted *p*-values were used to prioritize sensitivity. Interpretation was guided by effect sizes, biological plausibility, and internal consistency. For longitudinal cytokine and biomarker analyses, nominal two-sided *p*-values are reported without multiplicity correction, given the exploratory nature of the study, the modest sample size, and the limited number of low-dimensional prespecified outcome domains. These results are therefore interpreted as hypothesis-generating and evaluated in conjunction with effect size, confidence intervals, biological plausibility, and internal consistency rather than as multiplicity-controlled confirmatory findings. For high-dimensional transcriptomic and pathway analyses, multiple-comparison control was applied using Benjamini–Hochberg false discovery rate (FDR), as described below.

Sample sizes for each analysis reflect all available participants, samples, and assay results meeting prespecified quality-control criteria; no available data were excluded. Biological replicates were defined as independent participants, with longitudinal repeated measures modeled using subject-level random intercepts. Exact sample sizes for each model, assay, and time point are reported in Supplementary Data 4 and relevant figure legends.

### Single cell RNA sequencing

For scRNA-seq, cryopreserved CSF cells from each subject were thawed and processed simultaneously across all timepoints. Vials were rapidly warmed in a 37 °C water bath, after which 1.5 mL of pre-warmed X-VIVO 15 medium with Gentamicin and Phenol Red (Lonza, Cat# 418Q; hereafter referred to as *media*) was added to each cryovial. Cells were gently resuspended, transferred to a 15 mL Falcon tube containing 9 mL of pre-warmed media, and rinsed to recover cells. Tubes were centrifuged at 300 × *g* for 5 min at 4 °C, and the supernatant was removed. Cells were resuspended in 500 μL of media and transferred to a 1.5 mL tube; 30 μL was removed for viability and cell count assessment, as described above.

Most CSF samples were used for basal scRNA-seq. Four subjects with ≥24,000 viable cells per timepoint were selected for ex vivo stimulation followed by scRNA-seq. For each subject, CSF cells at each time point were evenly divided across three treatment conditions—media (control), lipopolysaccharide (LPS; 10 ng/mL), and heat-killed BCG (HKBCG; 1 × 10⁵ CFU/mL)—and stimulated for 4 h. To ensure matched stimulation density across all timepoints, we identified the timepoint with the lowest total cell count and used this value to define the number of cells per condition. Cells at each time point were divided evenly across three stimulation conditions at this fixed density. For timepoints with higher overall cell counts, cells were distributed across multiple wells per condition to maintain consistent density; these wells were pooled prior to 10× capture.

PBMCs from 3 of these 4 subjects were processed in parallel using the same stimulation conditions and matched input cell numbers. This single-cell PBMC stimulation was performed on separate aliquots and independently from the PBMC stimulations described above for cytokine release assays. All PBMC wells were pooled prior to sequencing to optimize 10x Genomics capture.

#### scRNA-seq sample processing

Following preparation, cells were centrifuged at 300 × *g* at 4 °C for 5 min and resuspended in run buffer (PBS + 1% BSA [Sigma Aldrich, Cat# A9576] and RNase inhibitor [Promega, Cat# N2615]) to a final concentration of 200–1500 cells/μL. Barcoding and library preparation were performed by Harvard’s Single Cell Core using the Chromium Next GEM Single Cell 5′ Reagent Kit v2 (Dual Index) (10× Genomics, CG000331). Briefly, cells were combined with barcoding master mix and loaded onto Chromium Next GEM Chip K with a target recovery of 500–10,000 cells per sample, followed by reverse transcription.

Gene expression (GEX) and T cell receptor (VDJ) libraries were prepared according to manufacturer's protocols. Library quality control (QC) was performed using Agilent TapeStation 4200 HS D1000 Screentape (Agilent Technologies). VDJ libraries were sequenced but are not analyzed in this manuscript. GEX and VDJ libraries were pooled separately based on target cell number and TapeStation molarity (200–1000 bp). Final pools were mixed in a 4:1 GEX:VDJ ratio and underwent qPCR-based quantification using the KAPA Library Quantification Kit (Roche). Shallow sequencing was initially performed on Illumina’s iSeq 100 v2 flow cell (26×10×10×90 cycles). Libraries were then rebalanced based on read depth and sequenced to target depth (50,000 reads/cell) on an Illumina NovaSeq 6000 S4 v1.5 flow cell using the same cycle scheme.

Reads were aligned to the GRCh38 (Ensembl 98) transcriptome (10× Genomics reference 2020-A) using Cell Ranger v6.0.2 with the multi-pipeline. Downstream data processing and analysis were conducted in R using the Seurat package (v4.3.0). Figures were generated using ggplot2^37^ in conjunction with Seurat.

#### scRNAseq quality control, cell identification, counts, and relative proportion

##### Software environment

All single-cell RNA-seq preprocessing, annotation, and visualization steps (except where noted) were performed in R (version 4.3.1). Analyses relied on the Seurat package (v4.3.0^38^) for preprocessing, clustering and reference-based cell annotation^39^; Harmony^40^ for batch integration; and scDblFinder^41^ for doublet detection. Visualization used ggplot2^37^ and patchwork^42^, and data wrangling was supported by the dplyr and tidyr packages^43^. Additional visualization and enrichment analyses were performed in R (ComplexUpset, ComplexHeatmap, clusterProfiler, org.Hs.eg.db) and Python (WordCloud), as detailed in the Transcriptional Response Classification and Visualization section. Statistical tests and transformations involving cell type proportions (e.g., Wilcoxon rank-sum tests, mixed-effects models) were conducted in Python or Stata, as detailed in the “Statistics and reproducibility” section.

##### Cell quality control

Cells were filtered based on the following criteria: total RNA counts per cell (nCount_RNA) > 1000, number of genes detected per cell (nFeature_RNA) > 750, log-transformed genes-per-UMI ratio (novelty) >0.8, and mitochondrial gene expression percentage (mitoRatio) between 0.75% and 12%. Mitochondrial content was calculated using the PercentageFeatureSet function, with mitochondrial genes identified by the prefix ^MT-. Novelty was defined as log₁₀(nFeature_RNA)/log₁₀(nCount_RNA). Filtering was applied uniformly across batches to ensure consistency. Doublets and multiplets were identified using the scDblFinder package^41^, filtered per 10× library (GEM batch), and excluded from downstream analyses.

##### Batch integration and cell identification

Following quality control, batches were merged using Seurat’s merge function and integrated using Harmony. Cell identities were assigned using Seurat’s FindTransferAnchors() and MapQuery() functions to map cells to a PBMC reference dataset, generating standardized L1-level annotations across CSF and PBMC samples.

##### Relative cell type proportions

Relative proportions were calculated per sample from L1 annotations. Baseline comparisons (e.g., CSF vs. PBMC, AD vs. non-AD) used nonparametric tests, and longitudinal changes (3 and 12 months versus baseline) were evaluated with mixed-effects models using a random intercept for donor. Statistical methods, including transformation of proportions and model specifications, are described in the Statistical Analysis section.

#### Pseudobulk modeling and differential expression analysis (DGE)

Two independent analyses were conducted to assess gene expression changes: one for basal-state CSF samples, and another for stimulated PBMC and CSF samples. In the basal-state analysis, pseudobulk aggregation was performed at the cell type × donor × timepoint level. For the stimulation analysis, aggregation was conducted at the cell type × donor × timepoint × treatment level. Ribosomal genes were removed to reduce technical noise. Differential gene expression (DGE) was performed using DESeq2^44^, which applied internal normalization to aggregated count data. Variance-stabilizing transformation was used for internal quality control, and principal component analysis and sample-level clustering were performed to assess outliers and batch consistency. These steps informed filtering decisions but were not used in downstream statistical modeling or visualizations. Covariates, including age, sex, and APOEε4 status were evaluated in exploratory models and showed minimal or inconsistent effects; they were excluded from final models.

#### Basal-state DGE (CSF only)

This analysis focused exclusively on CSF samples. Metadata included disease status (AD vs. non-AD), donor ID, and timepoint (0, 3, or 12 months post-BCG vaccination). A fixed-effects model was implemented in DESeq2 using the formula ~ disease + disease:donor + disease:time to account for individual variation nested within disease groups. Wald tests were used to assess within-group longitudinal changes (3 and 12 months vs. baseline) and cross-sectional differences by disease status at each time point. Interaction terms (disease × time) were explored but were not retained due to lack of significant differential expression.

#### Stimulated DGE (CSF and PBMC)

This analysis included both CSF and PBMC samples stimulated with control media (X-VIVO), lipopolysaccharide (LPS), or heat-killed BCG (HKBCG) at 0, 3, and 12 months. Metadata included donor ID, tissue type, treatment condition, and timepoint. Disease status was not included due to limited AD representation in CSF stimulation samples. Pseudobulk aggregation was performed at the level of cell type × donor × timepoint × treatment. A fixed-effects model was applied in DESeq2 using the formula ~ treatment_time, where the treatment_time factor represents combined treatment and timepoint conditions.

Analyses focused on stimulation effects within each time point by comparing each treatment condition to its corresponding control at the same time point. Contrasts included, for example, LPS_0mo vs. Ctrl_0mo, LPS_3mo vs. Ctrl_3mo, and LPS_12mo vs. Ctrl_12mo, as well as HKBCG_0mo vs. Ctrl_0mo, HKBCG_3mo vs. Ctrl_3mo, and HKBCG_12mo vs. Ctrl_12mo. These comparisons were used to calculate stimulation-induced deltas while accounting for temporal changes in basal gene expression. Longitudinal comparisons within each treatment group (e.g., LPS_12mo vs. LPS_0mo) are reported for reference. To characterize shared and compartment-specific response patterns, delta (Δ) values were computed from log₂ fold changes and used to classify gene trajectories and signatures, as described below.

#### Transcriptional response classification and visualization

##### UpSet

To visualize overlap in transcriptional responses across timepoints and compartments, delta values (Δ) were used to classify genes as upregulated (Δ >0.5) or downregulated (Δ <−0.5) for each stimulation condition and tissue. Intersections were visualized using UpSet plots (ComplexUpset v1.3.3 in R^45^), highlighting shared and unique gene sets across CSF and PBMC at 3 and 12 months. Only intersections containing >50 genes were plotted, with select biologically relevant conditions (e.g., BCG-CSF) retained regardless of size.

##### Heatmap and response clustering

To identify temporal expression dynamics, Δ values were computed as log₂ fold change differences from baseline (e.g., the change of 3 months was calculated as Δ₃ = log₂FC₃ − log₂FC₀, where the log₂FC₃ and log₂FC₀ denote the log fold changes between treatment and control groups at month 3 and 0, respectively), thereby normalizing for control-group drift. Genes were stratified into eight response clusters based on Δ₃ and Δ₁₂ thresholds (|Δ| > 0.5), including sustained, early, late, and switch-type patterns (e.g., Early Up–Late Down). Genes showing minimal change across both timepoints (|Δ₃| <0.5 and |Δ₁₂| <0.5) were classified as “None” and excluded from heatmap visualization. Heatmaps were generated using ComplexHeatmap (v2.21.2^46^) in R.

##### Wordcloud for GO enrichment

Gene Ontology Biological Process (GO-BP) enrichment was performed on each response cluster using clusterProfiler (v4.6.2^47^), with HGNC symbols mapped to Entrez IDs via org.Hs.eg.db (v3.16.0^48^). Terms with adjusted *p* < 0.05, *q* < 0.2 were retained. Enriched terms were tokenized (i.e., split into component words), filtered, grouped by cluster identity, and scored by the mean −log₁₀(*p*.adjust) of the top three terms (with smallest adjusted *p*). Weighted word clouds were generated using the Python WordCloud package (v1.9.3^49^), where word size reflects enrichment strength and color indicates response cluster identity. These visualizations were used to interpret temporal and compartment-specific transcriptional programs induced by LPS and BCG stimulation.

#### Pathway enrichment and immune trajectory visualization

To characterize functional shifts in immune-related pathways over time and between disease states, gene set enrichment analysis (GSEA) was performed using the fgsea package (v1.24.0) in R. Genes were ranked by the logFC from DESeq2, and enrichment was assessed against Reactome gene sets from MSigDB (v7.5.1) using the fgsea() function with a gene set size filter of 50 to 500 genes. Pathways with adjusted *p*-values (*p*adj) <0.05 were considered significantly enriched. Normalized enrichment scores (NES) and *p*adj values were retained for downstream analysis.

Baseline (AD vs. non-AD) and post-BCG comparisons (12-month timepoint only) were aligned by pathway name to allow interpretation of enrichment patterns across conditions and timepoints. Results from 3 months were excluded due to a lack of significant enrichment in both disease groups. Pathways were assigned to six functional categories (e.g., “Innate Immune Signaling”, “Metabolism”) using keyword-based classification. Pathways that could not be confidently assigned were labeled “Other” (See Supplementary Data 6).

For visualization, scatterplots displayed NES from baseline comparisons on the *X*-axis and NES from 12-month BCG responses (AD subjects only) on the *Y*-axis. Pathways were colored by category, with “Other” terms shown in gray and rendered semi-transparently to reduce visual emphasis. This approach supported the interpretation of joint disease- and treatment-associated pathway trajectories; for example, pathways in the upper right quadrant represent signatures that were upregulated in AD at baseline and further enriched after BCG. These quadrant relationships were used descriptively and do not imply convergence with disease biology.

To summarize global pathway activation across compartments, heatmaps were constructed using the ComplexHeatmap package (v2.21.2 in R). Pathways included in the heatmap met significance criteria in AD at 12 months (*p*adj <0.05) and were assigned to a defined biological category. Corresponding NES values from non-AD subjects were also shown to illustrate pattern similarity despite a lack of statistical significance.

### Outcome reporting and alignment with study objectives

This study was designed as an exploratory investigation of the immunological and neurobiological effects of BCG vaccination in older adults with and without Alzheimer’s disease. Given the exploratory design, outcomes were not formally designated as primary or secondary but were aligned with protocol-defined objectives. Each trial was guided by a prospectively defined set of scientific objectives outlined in the IRB-approved protocols, which served as the basis for subsequent endpoint designation on ClinicalTrials.gov. Safety and tolerability outcomes were collected prospectively throughout the study and are reported in Supplementary Data 7.

The first trial, which enrolled cognitively unimpaired and mildly impaired older adults, outlined a primary objective “to determine the most promising biomarkers of inflammation and immune response and neurodegeneration in blood and CSF in terms of feasibility of measurement, technical specifications, and BCG response of assays.” Secondary objectives included evaluating cognitive changes over time (MoCA, RBANS, NPI-Q, FAQ) and assessing the safety and tolerability of BCG vaccination. The second trial, focused on individuals with MCI or mild-to-moderate AD, similarly defined its primary objectives as evaluating the safety and tolerability of BCG and assessing immunological and AD-relevant biomarker changes in blood and CSF, along with exploratory neurocognitive endpoints. The sole secondary objective listed was a change in neuroimaging biomarkers (MRI) following BCG.

These protocol-level objectives informed the designation of clinical endpoints on ClinicalTrials.gov. All outcomes listed on ClinicalTrials.gov are reported in full, with the exception of several measures for which data were available from ≤4 participants, precluding meaningful group-level analysis or statistical modeling. These include (1) changes in structural and functional MRI (*n* = 3 participants with matched baseline and 12-month data), (2) CDR-SB and HADS cognitive and behavioral measures at 12 months (*n* = 4 each), and (3) DCT Clock Drawing Test (*n* = 3 at 12 months). These measures are not included in analyses.

Data for prespecified and registered outcomes are presented in full throughout the manuscript and associated Supplementary Data files. All statistical model outputs are reported in Supplementary Data 4, protocol-adjusted sensitivity analyses in Supplementary Data 5, and safety data in Supplementary Data 3.

## Results

### Study design, tolerability, and baseline characteristics

Between December 2020 and January 2024, 35 individuals were screened across two open-label clinical trials; 23 were enrolled and treated, while 12 did not meet eligibility criteria (Supplementary Fig. 1). Baseline characteristics of the enrolled cohort are presented in Supplementary Data 2, stratified by protocol. These 23 individuals compose the treated cohort included in all primary analyses, which assessed longitudinal immune and neurodegenerative biomarker changes following BCG immunotherapy. Key findings were consistent across both protocols, and inclusion of protocol as a covariate in sensitivity analyses did not materially affect observed effects. Sampling of CSF occurred at baseline, 3 months, and 12 months, while peripheral blood sampling occurred at baseline, 3 months, 6 months, and 12 months (Fig. 1A).

Participant groups were defined using a biomarker-based classification derived from k-means clustering of the CSF ptau181/Aβ42 ratio, yielding 11 non-AD and 12 AD participants; this stratification was supported by, but not dependent on, cognitive performance as assessed by MoCA (Supplementary Fig. 2, and Supplementary Data 3). All reported analyses reflect this exploratory framework, with prespecified and post hoc analyses distinguished as described in the Statistical Analysis section.

Follow-up and retention: 23 participants completed the 3-month visit, 20 completed 6 months, and 17 (74%) completed the full 12-month protocol. Detailed screen failures and attrition are summarized in Supplementary Fig. 1. Reasons for discontinuation included inability to complete procedures, personal reasons, and disease progression. BCG immunotherapy was well tolerated under prospective safety monitoring. One non-serious case of injection site dermatitis was reported. No other adverse events were attributed to BCG. Four cases of COVID-19 and two non-COVID respiratory infections occurred during the study period but were not considered related to study procedures (Supplementary Data 7). All participants developed a visible BCG scar, consistent with the expected local vaccine take.

### BCG induces robust trained immunity responses in peripheral blood mononuclear cells

To establish a reference for trained immunity induction (schematized in Fig. 1B), we first evaluated stimulus-specific cytokine responses in peripheral blood mononuclear cells (PBMCs), using ex vivo stimulation with heat-killed BCG (HKBCG) to measure IFN-γ production, and with lipopolysaccharide (LPS) and heat-killed *Candida albicans* (HKCA) to assess IL-1β, IL-6, and TNF-α responses. BCG immunotherapy was followed by strong peripheral cytokine recall responses across stimuli and timepoints.

At baseline, IFN-γ responses to HKBCG stimulation were modestly but significantly higher in non-AD individuals (Supplementary Data 3). Following BCG immunotherapy, both groups exhibited robust IFN-γ induction relative to baseline that persisted through 12 months. Non-AD participants showed an early peak at 3 months, while AD participants exhibited a delayed but sustained response. A significant group × time interaction was observed at 3 months, though overall response magnitudes converged by 12 months (Fig. 1C; source data provided; full model results in Supplementary Data 4).

LPS stimulation induced strong and sustained increases in IL-1β, IL-6, and TNF-α in PBMCs (Fig. 1D), with all cytokines peaking at 6 months in the overall model. No significant group differences were observed at baseline (Supplementary Data 3), and within-group increases from baseline were statistically significant at both 6 and 12 months, supporting a sustained trained immunity response in PBMCs. While no significant AD × time interactions were detected, response trajectories suggest delayed and sustained cytokine elevation in the AD group—particularly for IL-6 and TNF-α—compared to an earlier peak and partial resolution in non-AD participants. Full model results are provided in Supplementary Data 4. Inclusion of protocol as a fixed effect in *post hoc* sensitivity analyses did not materially alter the magnitude or significance of longitudinal findings across stimulation assays, clinical outcomes, cytokines, or biomarker analyses (Supplementary Data 5). Stimulation with HKCA also induced peak IL-1β and IL-6 responses at 6 months, consistent with a trained immunity profile, although responses were lower in magnitude than with LPS, and TNF-α did not reach statistical significance (Supplementary Fig. 3).

### Single-cell profiling and validation of immune cell composition

To characterize cell-type-specific responses, we performed single-cell RNA sequencing on longitudinal PBMC and CSF samples, yielding 254,292 high-quality cells (106,224 CSF; 148,068 PBMC). Cells were annotated using Label Transfer reference mapping and integrated with Harmony (see “Methods”), enabling joint clustering and visualization across compartments. The resulting UMAP embedding demonstrated successful integration and clear separation of canonical immune lineages, including monocytes, CD4⁺ and CD8⁺ T cells, NK cells, B cells, dendritic cells (DCs), and others (Supplementary Fig. 4a).

As expected, relative cell type abundances differed by compartment: NK and B cells were enriched in PBMCs (6.7% and 6.4%, respectively) compared to CSF (1.6% and 0.7%), while monocytes, CD8⁺ T cells, and DCs were more prevalent in CSF (Supplementary Fig. 4b, c). Baseline and longitudinal proportions are detailed in Supplementary Data 8 and 9. Within the CSF compartment, cell type proportions showed no consistent or sustained longitudinal changes over time or between AD and non-AD participants. At baseline, NK and DC proportions were modestly but significantly higher in AD participants (Supplementary Fig. 4d). CSF cell composition did not change meaningfully over the 1-year period following initial BCG treatment, aside from transient significant increases in B cells at 3 months and NK cells at 3 and 12 months. However, both are rare cell types in CSF—comprising only 0.7% and 1.6% of total cells, respectively—and thus more prone to sampling variability and overinterpretation (Supplementary Fig. 4e; see Supplementary Data 4 for MMRM results).

These findings confirm expected compartmental differences and stable CSF cellular composition over time. Given that downstream transcriptional analyses were conducted using pseudobulk methods within defined cell types, results reflect gene expression changes, not shifts in cell proportions.

Monocytes were the most abundant innate immune population in CSF and the dominant contributors to cytokine recall responses in PBMCs, prompting focused analysis of their transcriptional reprogramming over time.

### Trained immunity signatures and compartment-specific recall in CSF monocytes

Given their abundance in CSF and key role in cytokine responses, we next focused on monocytes to evaluate how BCG immunotherapy reprograms innate transcriptional responses over time. Using matched PBMC and CSF samples collected at baseline, 3 months, and 12 months, we assessed monocyte-specific responses to LPS and HKBCG stimulation. Samples were stimulated with LPS or heat-killed BCG (HKBCG) for 4 h prior to single-cell RNA sequencing. Log₂ fold-change (log₂FC) values were calculated as the ratio of stimulated (LPS or HKBCG) to unstimulated (media-only) gene expression. Results at all timepoints are presented as change from baseline (Δlog₂FC—see “Methods”).

**Fig. 2:**
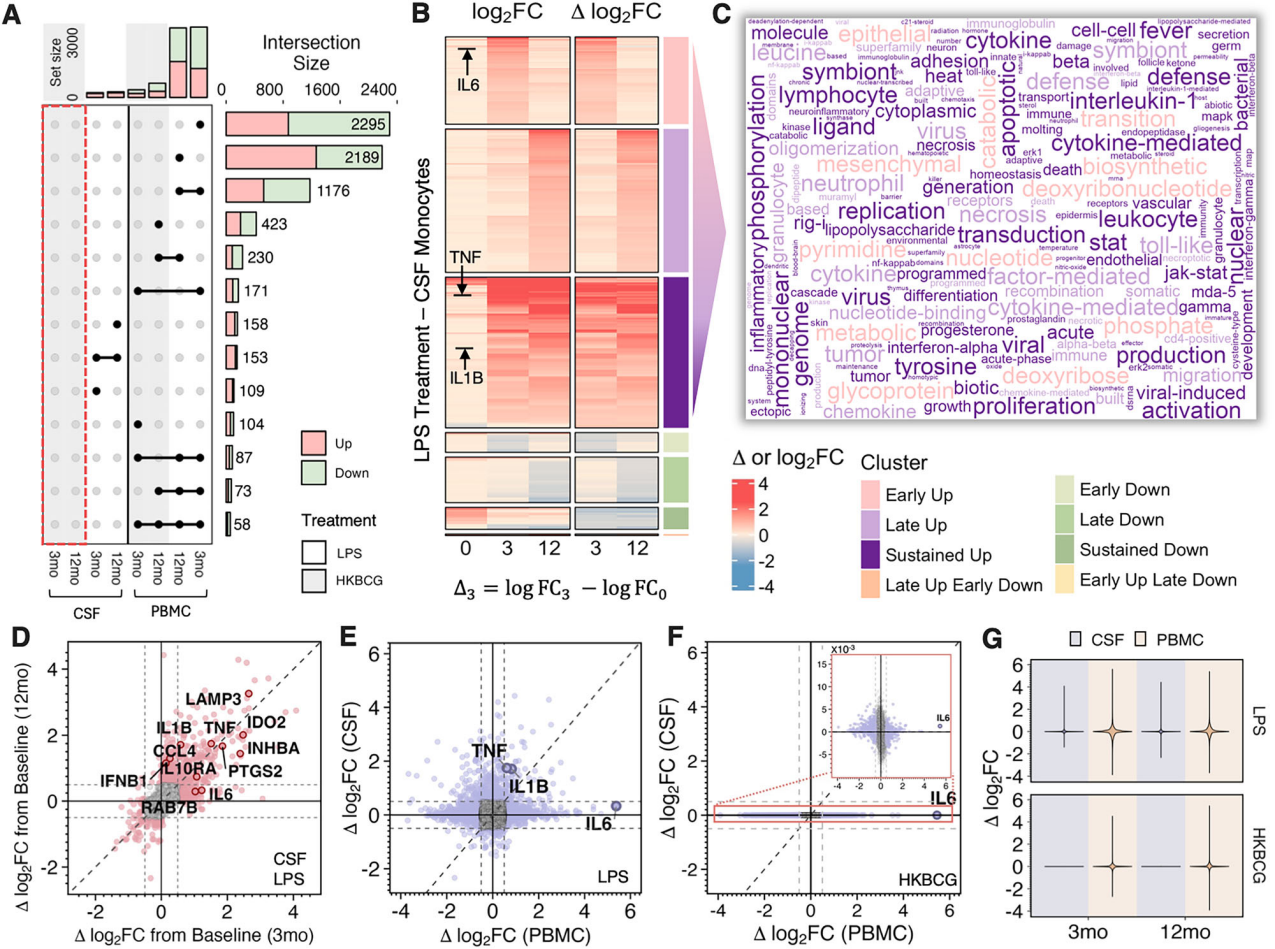
BCG induces compartment-specific transcriptional training in human monocytes. **A** Gene set intersections across monocytes from peripheral blood (PBMCs) and cerebrospinal fluid (CSF), stimulated ex vivo with LPS or heat-killed BCG (HKBCG) at 3 and 12 months post-BCG. UpSet plot shows differentially expressed genes (DEGs) across conditions, defined by Δ log₂FC ≥ ± 0.5. LPS induced sustained transcriptional changes in both PBMC and CSF monocytes, consistent with trained immunity, while HKBCG responses were restricted to PBMCs. **B** Heatmap of gene expression in CSF monocytes following LPS stimulation, showing log₂ fold change (FC) relative to media-only controls at baseline, 3, and 12 months (left), and Δ log₂FC from baseline at post-treatment timepoints (right). Eight clusters reflect distinct temporal patterns of up- and downregulation, including early, late, and sustained responses. **C** Word cloud of Gene Ontology Biological Process terms enriched in Early Up, Late Up, and Sustained Up clusters. Word size reflects term frequency. **D** Scatterplot of Δ log₂FC from baseline in CSF monocytes following LPS stimulation at 3 vs. 12 months. Genes such as IL1B, TNF, CCL4, and LAMP3 exhibit consistent upregulation across timepoints, indicating sustained trained immunity. Dashed lines at ±0.5 Δ log₂FC and the *y* = *x* diagonal provide visual reference points for magnitude and concordance of change. **E** Comparison of Δ log₂FC between PBMC and CSF monocytes at 12 months post-LPS stimulation. Genes including IL1B, IL6, and TNF show transcriptional training across compartments. **F** Comparison of Δ log₂FC between PBMC and CSF monocytes at 12 months following HKBCG stimulation. PBMCs exhibit strong transcriptional induction (e.g., IL6, LAMP3), while CSF monocytes show no meaningful response. An inset plot zooms in on the CSF *y*-axis (−0.01 to +0.015) to highlight the near-zero Δ log₂FC values; most points fall within the ±0.005 range, indicating a compartment-specific lack of transcriptional activation in CSF. **G** Violin plots of Δ log₂FC for LPS and HKBCG stimulation at 3 and 12 months. LPS responses are robust in both compartments; HKBCG responses are evident only in PBMCs. scRNA-seq stimulation analyses were performed in CSF monocytes from *n* = 4 donors and in PBMC monocytes from *n* = 3 donors; analyses are pooled across donors and not stratified by AD status due to limited sample size.

Figure 2A shows the overlap of differentially expressed genes (DEGs) in monocytes across compartments and stimuli. LPS stimulation elicited robust gene induction in both PBMC and CSF monocytes at 3 and 12 months, consistent with sustained stimulus-evoked responsiveness. Although PBMC monocytes exhibited the highest DEG counts—suggesting greater overall responsiveness—CSF monocytes also mounted a substantial transcriptional response to LPS at both timepoints. In contrast, HKBCG stimulation resulted in strong transcriptional activation in PBMC monocytes only, with CSF monocytes showing a negligible response. All stimulations were performed at matched cell density across timepoints within each subject, supporting that the observed differences reflect intrinsic compartmental biology rather than technical variability. Comprehensive DEG results for all cell types and stimulation conditions are provided in Supplementary Data 10.

Figure 2B displays LPS-induced gene expression profiles in CSF monocytes, highlighting the timing and persistence of trained immunity signatures following BCG immunotherapy. Genes with Δlog₂FC > 0.5 were clustered based on their longitudinal pattern, revealing early (3-month only), late (12-month only), and sustained (both) up- or down-regulation. Canonical trained immunity targets showed distinct timing: IL-6 was early-up, while IL1B and TNFA were sustained-up. Early-up genes also included HSD11B1, GAB3, RAB7B, and ITPKC, consistent with metabolic remodeling and immune signaling. Sustained-up genes included SERPINB2, LAMP3, and AIM2, implicating persistent stress and cytokine regulation. In contrast, late-up genes such as OAS1, ISG15, and CXCL10 suggested delayed increase of interferon signaling and leukocyte recruitment. These patterns reveal temporally distinct immune reprogramming in CSF monocytes, with early shifts in metabolism and signaling, followed by sustained inflammatory tone and delayed antiviral or chemokine responses.

Figure 2C displays a frequency-weighted word cloud of gene ontology terms enriched within each cluster of upregulated genes. Sustained upregulation (dark purple) was enriched for cytokine signaling, interferon responses, and viral defense pathways, including cytokine-mediated signaling, STAT/JAK signaling, and pattern recognition receptor signaling, consistent with persistent activation of innate immune programs. Early-only genes (peach/pink), upregulated at 3 months, were associated with pyrimidine catabolism, glycoprotein biosynthesis, and epithelial–mesenchymal transition, indicating transient engagement of nucleotide metabolism and structural remodeling. In contrast, late-only upregulation (light purple), detected only at 12 months, showed enrichment for toll-like receptor signaling, TNF pathways, neutrophil migration, and adaptive immune activation, suggesting progressive expansion of inflammatory and immune recruitment pathways over time. Together, these data suggest that BCG immunotherapy induces temporally structured transcriptional programs in CSF monocytes, with distinct early, late, and sustained phases. Full Δlog₂FC values, gene-level response data, and the GO term enrichment source data for these clusters are included in Supplementary Data 11.

Figure 2D identifies genes induced by LPS stimulation at both 3 and 12 months in CSF monocytes. Trained immunity–associated genes—such as PTGS2, IFNB1, IDO2, and LAMP3—were among those consistently upregulated across timepoints, supporting the presence of a durable, memory-like innate immune response.

Figure 2E–G compares stimulation responses across compartments. LPS elicited broadly similar trained immunity-associated transcriptional profiles in PBMC and CSF monocytes, including overlapping induction of canonical targets such as IL-1β, IL-6, and TNF-α, though with some differences in magnitude (Fig. 2E). In contrast, HKBCG stimulation triggered robust gene upregulation in PBMC monocytes—including >5 log₂FC increases in genes such as IL6—but failed to induce measurable responses in CSF monocytes (Fig. 2F). This lack of response is further illustrated in Fig. 2G, where violin plots show consistently flat transcriptional profiles in CSF at both 3 and 12 months, despite strong responses in PBMCs.

In CSF CD4⁺ and CD8⁺ T cells, LPS stimulation produced almost no gene induction at 4 h (Supplementary Fig. 5); only two genes passed the Δlog₂FC > 0.5 threshold in CD4⁺ cells, and none in CD8⁺. In PBMCs, monocytes showed broad and clustered transcriptional changes to LPS stimulation across timepoints, consistent with trained immunity. PBMC CD4⁺ and CD8⁺ T cells exhibited a distinct early-up signature at 3 months but no evidence of sustained activation at 12 months, suggesting a transient, possibly antigen-driven lymphoid response. These gene-level changes and cluster annotations, as well as full Δlog₂FC values and GO enrichment data, are available in Supplementary Data 11.

Together, these findings, derived from pooled analyses across participants, demonstrate that LPS stimulation reveals sustained BCG-associated transcriptional changes in CSF and PBMC monocytes, with sustained gene induction most clearly evident in the monocyte lineage. Stimulation assays with single-cell RNA sequencing were not performed at sufficient depth across AD and non-AD participants to support formal group comparisons; therefore, results reflect pooled analysis. Notably, despite being the immunizing agent, HKBCG failed to induce measurable transcriptional responses in CSF monocytes, in contrast to the robust responses seen in PBMCs, indicating compartment-specific constraints on stimulus-specific recall rather than a global failure of immune training. These findings suggest that peripheral immune training may not extend to direct stimulus-specific recall in CNS-associated innate cells, while preserving increased responsiveness to secondary inflammatory cues.

### BCG immunotherapy modulates CNS-associated immune pathways

**Fig. 3:**
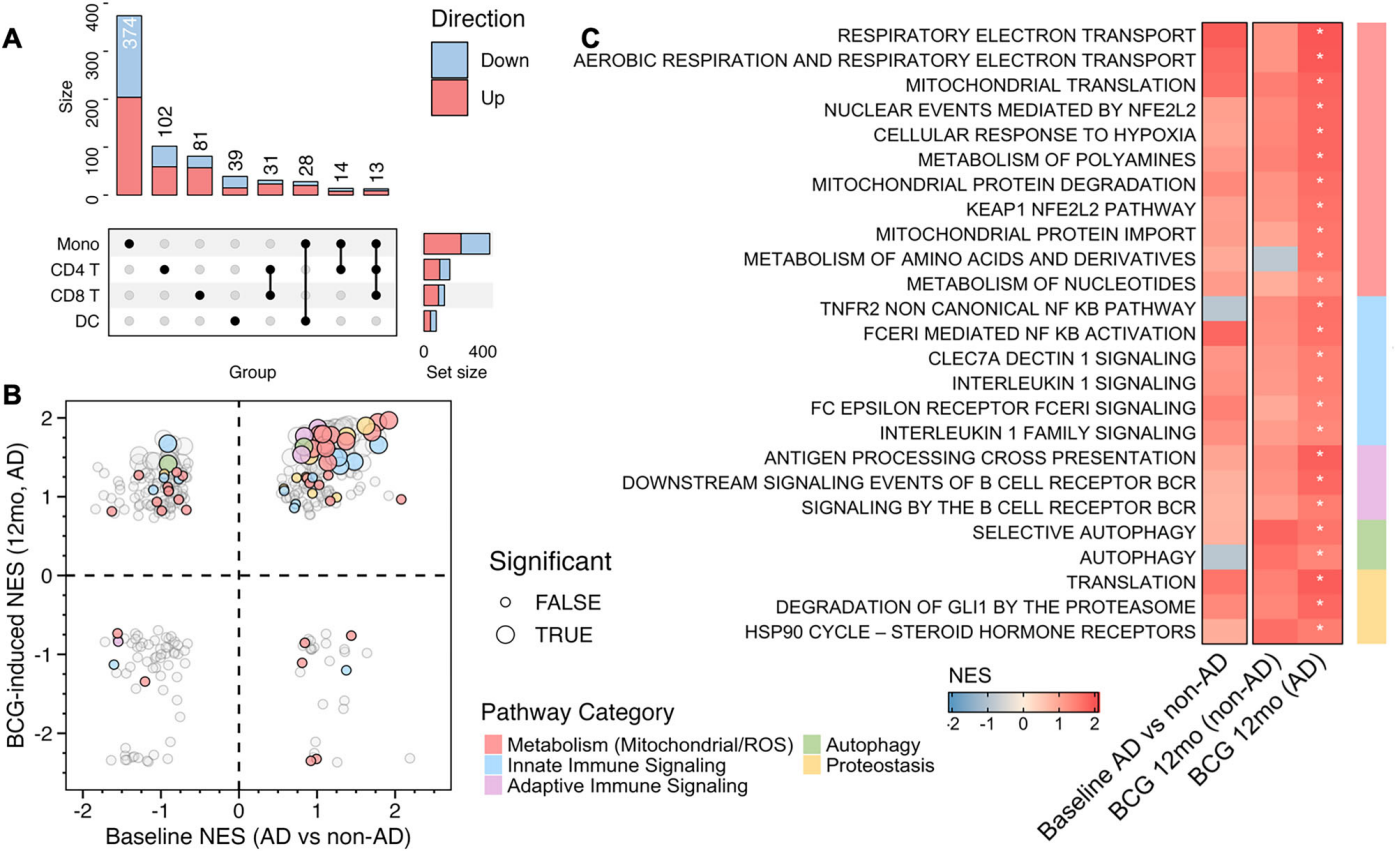
BCG immunotherapy alters basal immune-metabolic pathways in CSF monocytes. **A** Differential gene expression (DEG) analysis of cerebrospinal fluid (CSF) immune cells comparing AD vs. non-AD participants at baseline. The UpSet plot displays DEG intersections across cell types with ≥10 DEGs. Monocytes showed the largest DEG set, followed by CD4⁺ T cells, CD8⁺ T cells, and dendritic cells (DCs). **B** Gene set enrichment analysis (GSEA) of curated immune and metabolic pathways in CSF monocytes. The scatterplot shows normalized enrichment scores (NES) for each pathway based on baseline AD vs. non-AD (*x*-axis) and 12-month BCG-induced change within AD (*y*-axis). All pathways are color-coded by functional category; significant BCG-induced pathways (FDR-adjusted, two-sided *q* < 0.05) are shown as larger bubbles. Significant pathways fell in both the upper left and upper right quadrants, with a predominance in the upper right. **C** Heatmap of NES values across three comparisons in CSF monocytes: baseline AD vs. non-AD, 12-month change in AD, and 12-month change in non-AD. Pathway categories match those in **B**, and all significant pathways not classified as “other” are shown. Many pathways enriched at baseline in AD also changed significantly after BCG, including those related to mitochondrial metabolism, cytokine and interleukin signaling, antigen presentation, and proteostasis. Non-AD participants showed directionally similar, though non-significant, patterns, supporting a BCG-driven effect independent of disease status. All enrichment scores were derived from GSEA using log₂ fold changes as input. Full gene-level and pathway enrichment results are in Supplementary Data 6; equivalent analyses for CD4⁺ T cells are shown in Supplementary Fig. 6.

We next examined whether BCG immunotherapy alters the unstimulated (basal) transcriptional landscape of CSF immune cells at 12 months post-treatment, and whether these effects differ between AD and non-AD participants. Figure 3A shows the number of DEGs at baseline between AD and non-AD participants across major CSF immune cell types. Monocytes exhibited the most pronounced differences, followed by CD4⁺ and CD8⁺ T cells. Full DEG results across all CSF cell types are provided in Supplementary Data 12.

To assess functional consequences of these differences, we performed GSEA using curated immune and metabolic pathways. Figure 3B plots enrichment scores for each pathway based on two comparisons: baseline differences between AD and non-AD participants (*x*-axis) and BCG-induced changes at 12 months within AD participants (*y*-axis). Significant 12-month pathways are shown as large bubbles and categorized by function, including metabolism, innate and adaptive immune signaling, autophagy, and proteostasis. Enrichment patterns in non-AD participants followed similar directions, though they did not reach significance. The complete pathway enrichment results are available in Supplementary Data 6.

Figure 3C presents a heatmap of NES for CSF monocytes across three comparisons: baseline AD vs. non-AD, and BCG-induced changes at 12 months in AD and non-AD participants separately. BCG treatment in AD enriched multiple immune-metabolic pathways, including aerobic respiration and respiratory electron transport, interleukin-1 family signaling, and TNFR2-related NF-κB signaling. While effects were strongest in AD, non-AD participants exhibited similar directional shifts. Notably, both TNFR2 non-canonical NFᴋB signaling and autophagy were suppressed at baseline in AD, suggesting that BCG may restore access to key immune-regulatory programs disrupted in disease.

CD4⁺ T cells demonstrated similar findings (Supplementary Fig. 6). BCG significantly enriched immune-metabolic pathways in AD participants, including several that were downregulated in AD at baseline—such as FCεRI-mediated NF-κB activation, antigen processing and cross-presentation, and the HSP90 steroid hormone receptor cycle. Directionally similar but nonsignificant changes were observed in non-AD CD4⁺ cells. CD8⁺ T cells did not exhibit significant pathway enrichment at any time point.

These results show that BCG immunotherapy is associated with sustained transcriptional changes in CSF monocytes and CD4⁺ T cells, affecting a range of metabolic and immunoregulatory pathways. Directionally similar changes were observed in AD and non-AD individuals, echoing the shared LPS transcriptional signatures observed earlier.

### BCG induces divergent plasma and convergent CSF cytokine responses

At baseline, plasma and CSF cytokine levels did not differ significantly between AD and non-AD participants (Supplementary Data 3), providing a uniform starting point to evaluate longitudinal immunotherapy-related cytokine changes.

**Fig. 4:**
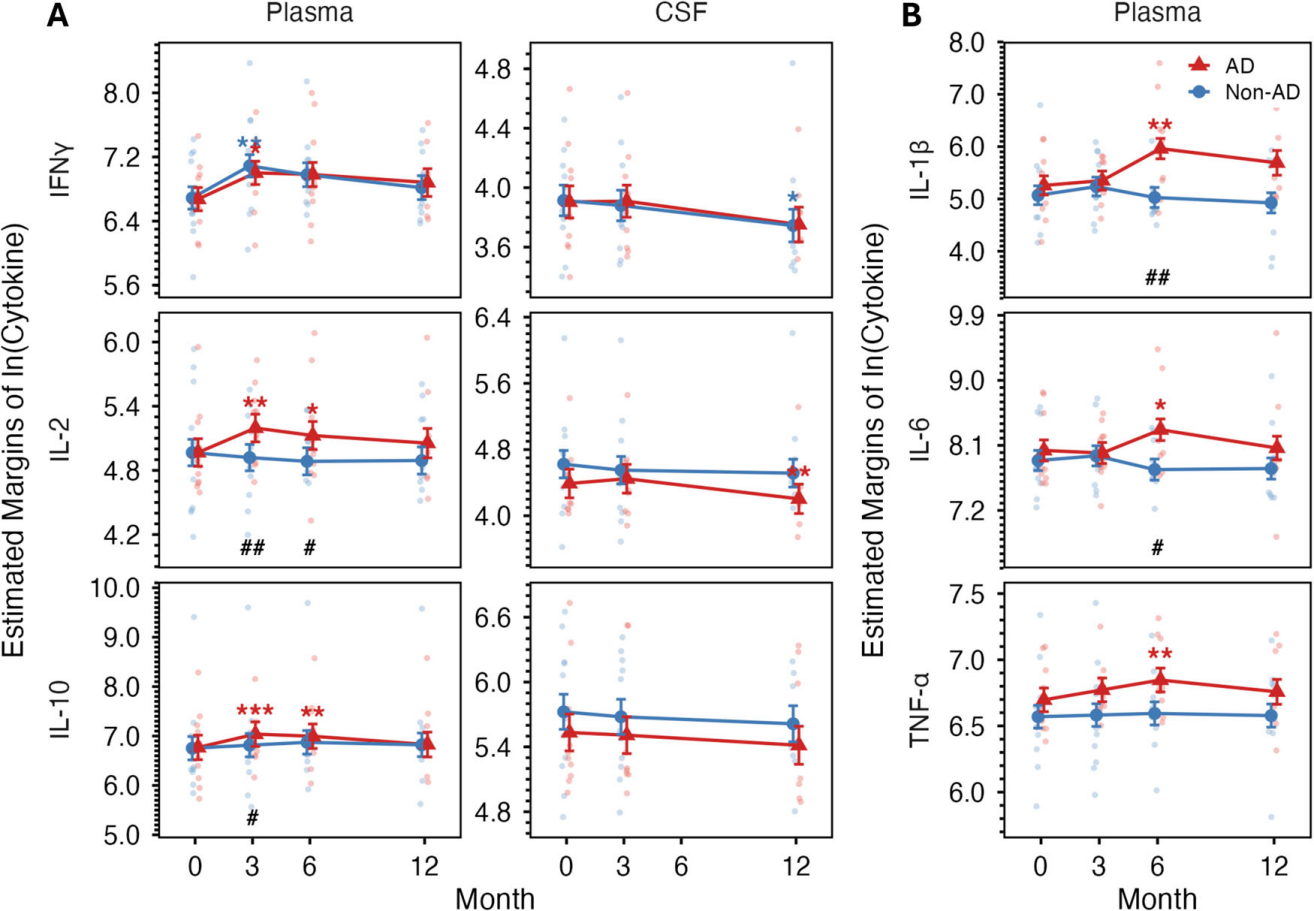
Cytokine responses to BCG immunotherapy differ by compartment and AD status. **A** Longitudinal changes in IFNγ, IL-2, and IL-10 following BCG vaccination are shown for plasma (left) and CSF (center), modeled separately for AD and non-AD participants. IFNγ increased significantly in plasma in both groups and decreased in CSF at 12 months. Plasma IL-2 and IL-10 rose selectively in AD participants, while CSF IL-2 declined over time, particularly in AD. CSF IL-10 showed a non-significant downward trend in both groups. **B** Plasma levels of IL-1β, IL-6, and TNF-α increased in AD participants over time, while remaining stable in non-AD participants. Lines represent estimated marginal means ± SEM from linear mixed-effects models including Month, AD status, and their interaction where supported. When no interaction was retained, plotted group-stratified estimates reflect the same pooled time effect and should not be interpreted as evidence of group differences. Asterisks denote within-group significance compared to baseline (^*^p < 0.05, ^**^p < 0.01, ^***^p < 0.001). Hash marks indicate significant AD × Month interaction (^#^*p* < 0.05, ^##^*p* < 0.01). Apparent differences between AD and non-AD trajectories should be interpreted in light of this modeling approach. For example, CSF IFNγ and IL-2 showed significant overall decreases at 12 months in the pooled model, though significance is displayed only for one group due to stratified graphing. Exact annotated *p* values for within-group comparisons versus baseline were: plasma IFN-γ AD (3m *p* = 0.037), non-AD (3m *p* = 0.008); CSF IFN-γ non-AD (12m *p* = 0.049); plasma IL-2 AD (3m *p* = 0.003, 6m *p* = 0.045); CSF IL-2 non-AD (12m *p* = 0.009); plasma IL-10 AD (3m *p* < 0.001, 6m *p* = 0.003); plasma IL-1β AD (6m *p* = 0.001); plasma IL-6 AD (6m *p* = 0.022); plasma TNF-α AD (6m *p* = 0.002). Exact annotated AD × Month interaction *p* values were: plasma IL-2 (3m *p* = 0.009, 6m *p* = 0.031), plasma IL-10 (3m *p* = 0.038), plasma IL-1β (6m *p* = 0.009), and plasma IL-6 (6m *p* = 0.019). Baseline levels did not differ significantly between groups for any cytokine. Sample sizes vary by analyte and timepoint; full model Ns, effect estimates, 95% confidence intervals, and exact *p* values are reported in Supplementary Data 4. All statistical tests were two-sided; *p* values are nominal and not adjusted for multiple comparisons, consistent with the exploratory design.

Following BCG immunotherapy, resting (basal) cytokine concentrations changed over time in both plasma and CSF. These effects reflect systemic and CNS immunomodulation and were further evaluated by AD status. In plasma, IFN-γ increased in both groups at 3 and 6 months before returning to baseline, with no significant AD × Time interaction. In contrast, IL-2 and IL-10 rose selectively in AD participants, peaking early, while remaining stable in non-AD (Fig. 4A).

These early AD-specific elevations were followed by a delayed rise in pro-inflammatory cytokines—IL-1β, IL-6, and TNF-α—which also increased significantly in AD but not in non-AD participants (Fig. 4B).

In CSF, cytokines followed a shared, immunoregulatory trajectory across groups. IFN-γ and IL-2 declined significantly by 12 months (*p* = 0.010 and *p* = 0.006, respectively), with IL-10 showing a similar trend (Fig. 4A). Consistent with our prespecified analytic approach (see “Methods”), no significant AD × Time interactions were detected, so we focused on the overall effects of time across participants. Inflammatory cytokines such as IL-1β, IL-6, and TNF-α remained low and did not show consistent longitudinal changes. Full statistical results are provided in Supplementary Data 4.

These findings underscore a compartment-specific immune signature of BCG immunotherapy: plasma cytokine responses were disease-modulated, with greater and more phased responses in AD participants, whereas CSF cytokine profiles followed a shared downregulatory trajectory independent of AD status.

### Amyloid-β dynamics differ by AD status following BCG immunotherapy

At baseline, AD participants showed significantly lower CSF Aβ42/40 ratios and Aβ42 levels, and higher ptau181 and ptau181/Aβ42 ratios compared to non-AD participants (all *p* < 0.001), consistent with established biomarker criteria. Plasma Aβ42/40 ratios were also reduced in the AD group (Supplementary Data 3).

**Fig. 5:**
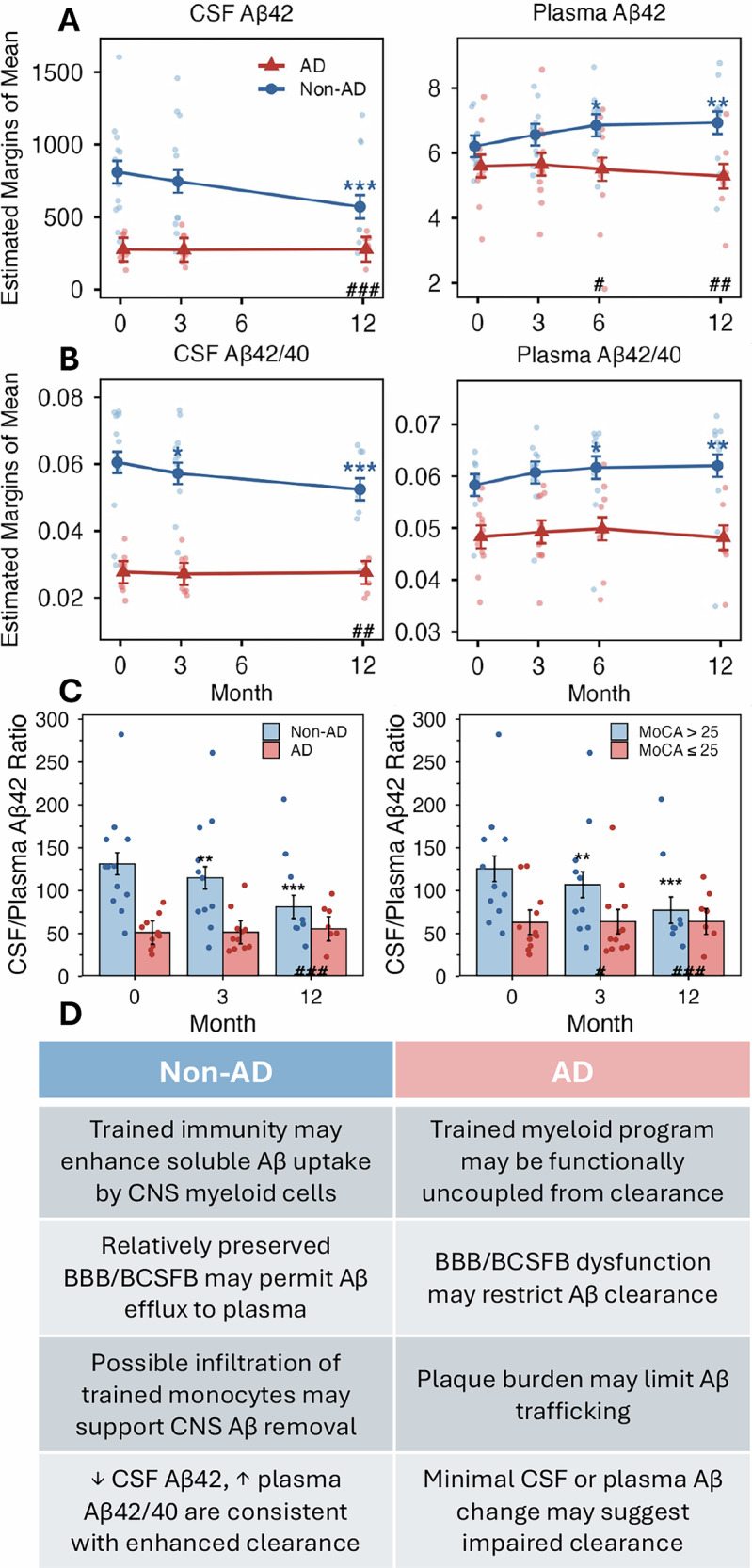
Amyloid-β dynamics diverge by AD status following BCG immunotherapy. **A**, **B** In non-AD participants, CSF Aβ42 and Aβ42/40 ratios significantly declined over time, while plasma Aβ42 and Aβ42/40 ratios significantly increased. No significant changes were observed in AD participants. **C** The CSF-to-plasma Aβ42 ratio declined significantly by 3 months and further by 12 months in both classification approaches: by 38.2% in biomarker-defined non-AD participants (left) and 38.6% in participants with MoCA > 25 (right). **D** Conceptual, hypothesis-generating model summarizing mechanisms that may underlie divergent Aβ dynamics across disease stage, including immune-associated processes in non-AD and impaired trafficking or sequestration in AD. Data are estimated marginal means ± SEM from linear mixed-effects models including time-only, AD × Month, or MoCA × Month terms as appropriate. Asterisks denote within-group significance compared to baseline (^*^p < 0.05, ^**^p < 0.01, ^***^p < 0.001). Hash marks indicate significant AD × Month interaction (^#^*p* < 0.05, ^##^*p* < 0.01, ^###^*p* < 0.001). Exact annotated *p* values for within-group comparisons versus baseline were: CSF Aβ42 non-AD (12m *p* < 0.001); plasma Aβ42 non-AD (6m *p* = 0.011, 12m *p* = 0.004); CSF Aβ42/40 non-AD (3m *p* = 0.020, 12m *p* < 0.001); plasma Aβ42/40 non-AD (6m *p* = 0.020, 12m *p* = 0.009); CSF/plasma Aβ42 ratio non-AD (3m *p* = 0.009, 12m *p* < 0.001); and in the MoCA-based sensitivity model, CSF/plasma Aβ42 ratio for MoCA >25 (3m *p* = 0.008, 12m *p* < 0.001). Exact annotated interaction *p* values were: CSF Aβ42 AD × Month (12m *p* < 0.001); plasma Aβ42 AD × Month (6m *p* = 0.039, 12m *p* = 0.007); CSF Aβ42/40 AD × Month (12m *p* = 0.001); CSF/plasma Aβ42 ratio AD × Month (12m *p* < 0.001); and in the MoCA-based sensitivity model, MoCA × Month interactions at 3m (*p* = 0.042) and 12m (*p* < 0.001). BBB denotes the blood-brain barrier, and BCSFB denotes the blood-cerebrospinal fluid barrier. Sample sizes vary by analyte and timepoint; full model Ns, effect estimates, 95% confidence intervals, and exact *p* values are reported in Supplementary Data 4. All statistical tests were two-sided; *p* values are nominal and not adjusted for multiple comparisons, consistent with the exploratory design.

We next assessed longitudinal changes in amyloid-β following BCG immunotherapy. Over the 12-month period, amyloid-β dynamics diverged by disease status, with changes observed only in non-AD participants. In non-AD participants, CSF Aβ42 levels declined significantly, along with reductions in Aβ42/40 ratios (Fig. 5A, B). In parallel, plasma Aβ42 increased at 6 and 12 months, a pattern not observed in AD participants. These findings were supported by significant AD × Time interactions, indicating a disease-specific divergence in Aβ trajectories. Individual participant trajectories are shown in Supplementary Fig. 7.

To assess net directional shifts in Aβ distribution, we examined the CSF-to-plasma Aβ42 ratio, which declined significantly by 3 months and decreased by 38.2% over 12 months in non-AD participants, with no meaningful change in the AD group (Fig. 5C, left). A similar pattern was observed when stratifying participants by MoCA score, with individuals scoring >25 showing a significant decline at 3 months and a 38.6% reduction at 12 months (Fig. 5C, right). These findings demonstrate consistent changes in CSF and plasma Aβ42 measures in non-AD participants across both biomarker- and cognition-based classification approaches, supporting the robustness of the observed pattern.

No significant longitudinal changes were observed in CSF ptau181, NFL, or GFAP levels in either group over the 12-month period. Figure 5D outlines potential mechanisms underlying the observed changes in CSF and plasma Aβ42 in non-AD participants and is discussed further in the Discussion. Cognitive performance, assessed with the Repeatable Battery for the Assessment of Neuropsychological Status (RBANS), remained stable in AD participants over 12 months, with modest improvements observed in non-AD participants, likely reflecting test–retest effects (Supplementary Fig. 8).

Together, these results indicate that BCG immunotherapy is associated with trained immunity-like responses in CSF monocytes within the CNS compartment, characterized by increased transcriptional responsiveness, sustained immune–metabolic changes, and disease-stage–specific differences in AD-related biomarkers. While this study was not designed to assess clinical efficacy, the observed decreases in CSF Aβ42 alongside increases in plasma Aβ42 are descriptive findings and do not imply directionality of effect or clinical benefit.

## Discussion

This open-label study shows durable, compartment-specific immune remodeling after BCG immunotherapy and provides evidence consistent with trained immunity-like transcriptional recall in CSF immune cells accompanied by sustained metabolic pathway enrichment. This work outlines a conceptual framework—grounded in longitudinal CSF immune profiling, stimulation assays, and transcriptomics—that links peripheral immune training with CNS-associated immune responses and AD-relevant biological readouts in humans, without asserting clinical efficacy. Systemic BCG administration was associated with increased stimulus-evoked responses and transcriptional changes in CSF monocytes, along with sustained immune-metabolic pathway enrichment in both monocytes and CD4⁺ T cells at 12 months. These effects were paralleled by cytokine modulation, AD-relevant biomarkers, and upregulation of oxidative and mitochondrial metabolic pathways. Immune responses were compartmentalized: peripheral responses were more pronounced in AD participants, while CSF responses shifted toward an immunoregulatory state across groups. In non-AD participants, BCG was associated with changes in Aβ42 dynamics, characterized by decreases in CSF Aβ42 and increases in plasma Aβ42. Collectively, these findings suggest that BCG is associated with neuroimmune remodeling and warrants testing in controlled studies.

BCG was associated with a trained immunity-like phenotype in CSF monocytes, evidenced by increased transcriptional responsiveness to LPS and upregulation of genes linked to cytokine signaling, oxidative stress, and metabolic adaptation. Despite the absence of direct antigen recall to HKBCG, these cells exhibited sustained transcriptional and functional changes, suggesting that these CNS-associated changes may arise through secondary mechanisms such as systemic cytokine signaling or the recruitment of trained myeloid precursors. The magnitude and coherence of the pseudobulk response argue against a marginal or bystander population. One plausible mechanism involves bone marrow–derived progenitors that undergo peripheral training following BCG exposure and later migrate into the CNS, consistent with evidence that BCG reprograms hematopoietic progenitors and alters downstream myeloid function^50,51^. Alternatively, or additionally, BCG may epigenetically reprogram resident CNS myeloid cells without direct antigen exposure. In support of this, recent work in aged mice has shown that peripheral BCG vaccination enhances the reparative function of microglia via increased histone acetylation (H3K27ac, H3K4me3), restoring transcriptional accessibility and immune plasticity^52^. Together, these findings are consistent with a model in which BCG immunotherapy may reshape neuroimmune tone via trained immunity, with potential implications for CNS homeostasis and protein clearance^25,53^. CSF represents a CNS-accessible compartment, and these findings reflect changes in CSF monocytes; the contribution of peripheral monocyte trafficking into the CSF cannot be excluded.

Importantly, the trained immunity phenotype observed here reflects increased stimulus-evoked responsiveness rather than sustained basal inflammation. In the CNS, BCG-induced immune training was detectable primarily through transcriptional recall responses to LPS stimulation, while basal CSF cytokine levels remained low or declined over time. LPS was used as the primary recall stimulus because it provides the most sensitive and widely accepted functional readout of trained innate immunity in human monocytes, whereas heat-killed *Candida albicans* elicited qualitatively similar but lower-magnitude cytokine responses that did not alter overall interpretation (Supplementary Fig. 3). This distinction is particularly relevant in AD, where chronic, unregulated inflammation is pathogenic^1–5^, but preserved or restored immune responsiveness may support homeostatic functions such as debris sensing, stress adaptation, and coordinated immune–metabolic signaling. Trained immunity has been defined as a memory-like enhancement of innate immune responsiveness without constitutive inflammatory activation^11^, and recent CNS-focused work demonstrates that immune training can restore functional capacity in aged myeloid cells without inducing inflammatory gene expression^52,53^. Our data are consistent with this framework, in which immune training increases adaptive reserve rather than driving persistent neuroinflammation. Future studies incorporating epigenetic profiling (e.g., chromatin accessibility or histone modification analyses) will be required to determine whether disease-stage–specific epigenetic priming accounts for the divergent durability and functional coupling of trained immune responses observed between AD and non-AD participants. Consistent with Fig. 1D, peripheral cytokine responses resolved over time in non-AD participants but remained elevated in AD participants, indicating disease-stage-dependent differences in immune regulation. Sustained cytokine induction in AD may reflect disease-stage-dependent epigenetic or metabolic set points that preserve inflammatory responsiveness while limiting functional coupling to downstream clearance or regulatory pathways, a mechanism not directly tested here.

BCG was also associated with changes in Aβ dynamics and CNS cytokine profiles. In non-AD participants, BCG vaccination led to a decline in CSF Aβ42, an increase in plasma Aβ42, and a 38% reduction in the CSF-to-plasma Aβ42 ratio over 12 months. These findings are consistent with a pattern of decreased CSF Aβ42 and increased plasma Aβ42 in non-AD participants, which may reflect altered cross-compartment amyloid dynamics, although specific mechanisms such as redistribution or efflux cannot be determined from this study. These shifts were not observed in AD participants, consistent with the view that Aβ accumulation in AD reflects impaired clearance rather than increased production^54^. These findings align with a small prospective study reporting increased plasma Aβ42/40 ratios after BCG administration in healthy adults, particularly younger individuals^55^. While the study did not assess brain amyloid burden directly, the plasma findings are directionally aligned with our own. A conceptual, hypothesis-generating model summarizing possible contributors to these diverging trajectories is presented in Fig. 5D, including CNS-trained immunity, barrier function, and myeloid cell dynamics across disease states.

These Aβ shifts were accompanied by a distinct CNS cytokine profile: CSF IFN-γ and IL-2 declined, while IL-1β, IL-6, or TNF-α remained low, indicating a non-inflammatory shift in CNS immune tone. These changes were consistent across AD and non-AD participants, suggesting that BCG did not elicit central inflammation even in the presence of underlying pathology. This stands in contrast to the periphery, where AD participants mounted a stronger and more sustained cytokine response, including elevations in IL-2, IL-10, IL-1β, IL-6, and TNF-α. The divergence between peripheral and CNS responses highlights a compartmentalized immune trajectory shaped by disease state. In AD participants, the muted CNS cytokine response—despite underlying pathology—may reflect compartment-specific immune adaptation or exhaustion, as described in neurodegenerative disease contexts^9^.

While peripheral cytokine responses to BCG were more pronounced and sustained in AD participants, this does not necessarily indicate a beneficial trained immunity phenotype. Rather, it may reflect disease-stage–dependent immune dysregulation, in which inflammatory responsivity is preserved or exaggerated but not clearly aligned with downstream biomarker changes. In contrast to non-AD participants, who exhibited decreases in CSF Aβ42 alongside increases in plasma Aβ42, AD participants showed no comparable Aβ42 changes despite heightened cytokine induction. In Alzheimer’s disease, immune activation is often dissociated from effective protein clearance and has been linked to impaired phagocytic efficiency, metabolic inflexibility, and chronic inflammatory signaling^9,54^. Together, these findings highlight differences in how immune and biomarker signals relate across disease states.

Preclinical studies suggest that peripheral immune modulation can influence CNS immune dynamics and solute clearance. In transgenic AD mice, BCG immunization reduces neuroinflammation and facilitates Aβ clearance, partly by recruiting phagocytic macrophages^24,25^. The reduction in neuroinflammation may also result from BCG-induced expansion of Treg cells, mediated through adaptive immune training and epigenetic reprogramming of genes, including those regulating the T cell receptor^56^. A recent study showed that acute experimental colitis in 5⨯FAD mice elevated peripheral cytokines, disrupted blood-brain-barrier integrity, and triggered infiltration of inflammatory monocytes into the brain, ultimately reducing parenchymal Aβ plaque burden^57^. Although our study cannot distinguish resident from recruited myeloid cells in CSF, the convergence of immune activation, non-inflammatory CNS signaling, and altered Aβ dynamics in non-AD individuals supports a hypothesis-generating model in which peripheral immune training may contribute to CNS clearance without triggering inflammation.

These findings are further supported by recent work showing that innate immune training can restore functional homeostasis in aging brain myeloid cells. Tiwari et al.^52^ showed that BCG reprograms epigenetic accessibility in microglia without inducing inflammatory gene expression, enabling improved debris clearance and immune readiness. Together, these observations raise the possibility that BCG engages systemic immune pathways that recalibrate CNS immune tone and support early-stage protein clearance. Further work is needed to define the underlying mechanisms, including cytokine signaling, trained monocyte trafficking, and local immune adaptation at CNS interfaces.

More broadly, BCG immunotherapy revealed durable immune plasticity across compartments and cell types in older adults. While monocytes exhibited both sustained functional and transcriptional changes^11^, CNS lymphoid cells also showed basal transcriptional changes at 12 months, despite no measurable trained immunity response. These shifts may reflect delayed or metabolically driven adaptation, suggesting that long-term immune remodeling associated with BCG is not limited to the myeloid lineage. Although trained immunity is defined by enhanced responsiveness to secondary stimuli^51^, basal transcriptional remodeling—as seen here in lymphoid cells—may represent a distinct, slower-developing form of adaptive immune training. This interpretation aligns with prior work suggesting that durable metabolic reprogramming in lymphoid cells may take years to manifest fully^29^. These findings illustrate that immune remodeling remains accessible in aging and may be leveraged to support resilience before the onset of overt neurodegeneration^58^.

This study has several important limitations. This exploratory, open-label study included a modest and demographically limited sample, which may constrain generalizability and preclude causal inference regarding BCG-specific effects in the absence of a placebo control. While the use of CSF provides rare insight into CNS immune remodeling, replication in larger, placebo-controlled cohorts will be essential to validate these findings. Variability in cytokine measurements differed across analytes, with CSF IL-1β showing a higher coefficient of variation than other markers; however, all longitudinal samples from each participant were assayed on the same plate, preserving the reliability of within-subject comparisons, and key findings were supported by consistent patterns across multiple cytokines.

The durability of these effects beyond one year remains unknown, as does the extent to which observed CNS changes reflect reprogramming of resident cells versus recruitment of trained peripheral monocytes or T cells. Given emerging evidence that epigenetically trained adaptive immune responses—particularly involving T cells—may evolve over multi-year timescales and correlate with clinical outcomes^59^, longer-duration BCG trials may be necessary to fully capture their impact.

The magnitude of immune responses observed here may also reflect use of the Japan BCG strain (Tokyo 172), which is closely related to early-passage strains such as Pasteur and has demonstrated strong and lasting immune activation in prior studies^60–62^. Strain-specific immunogenicity should be carefully considered in future efforts to harness trained immunity in aging populations. Future studies should examine how innate and adaptive immune training, metabolic and epigenetic remodeling, and peripheral—CNS crosstalk evolve over time and influence resilience to neurodegeneration.

An additional unresolved question concerns which components of BCG are necessary and sufficient to drive the immune remodeling observed in this study. BCG is a complex live attenuated organism with well-described adjuvant properties, engaging multiple pattern-recognition receptors and inducing durable metabolic and epigenetic reprogramming of innate immune cells. Whether CNS immune effects require live organisms, persistent antigen exposure, or could be recapitulated by defined bacterial components or adjuvant formulations remains unknown. Addressing this question will be critical for translation, including the development of simplified, potentially oral or mucosal immune-modulatory strategies that could be deployed at scale in aging populations.

Epidemiologic studies have consistently reported reduced risk of AD and other dementias in individuals who received intravesical BCG, suggesting potential long-term benefits of immune modulation^18–21^. Complementary findings from recombinant Zoster vaccination also suggest immune-based protection against dementia^63^, reinforcing the relevance of peripheral immune modulation across vaccine platforms. Recent prospective findings further support this hypothesis, showing increased plasma Aβ42/40 following BCG in healthy individuals, particularly younger adults^55^.

Together, these findings suggest that trained immunity, inducible by a widely available vaccine, may modulate neuroimmune tone and promote resilience. Our results demonstrate immune remodeling and altered Aβ dynamics in older adults, particularly those without established AD pathology. This pattern suggests a potential window for early intervention before irreversible neurodegeneration occurs. However, the biomarker and immune effects observed here were confined to individuals without established Alzheimer’s disease and do not demonstrate prevention, disease modification, or clinical benefit; accordingly, these implications should be considered hypothesis-generating. The ability to reprogram CNS immune responses without inducing inflammation may be particularly relevant in aging, where systemic inflammation is a known risk factor for cognitive decline^1,2,4^. Larger, placebo-controlled studies will be required to determine whether these immune changes translate into meaningful clinical outcomes.

## Supplementary information

Supplementary Information

Description of Additional Supplementary Files

Supplementary Data 1

Supplementary Data 2

Supplementary Data 3

Supplementary Data 4

Supplementary Data 5

Supplementary Data 6

Supplementary Data 7

Supplementary Data 8

Supplementary Data 9

Supplementary Data 10

Supplementary Data 11

Supplementary Data 12

## Data Availability

Raw and processed single-cell RNA sequencing (scRNA-seq) data for basal cerebrospinal fluid (CSF) analyses are available in the NCBI Gene Expression Omnibus (GEO) under accession number GSE281606. Raw and processed scRNA-seq data for CSF cell and peripheral blood mononuclear cell (PBMC) ex vivo stimulation analyses are available under accession number GSE280720. Biomarker and neuropsychological data generated in this study are provided in Supplementary Data 1 (Source data). Source data underlying all main figures are provided as Supplementary Data files. Detailed descriptions of all Supplementary Data files are provided in the Supplementary Data Legends file. Additional de-identified data supporting the findings of this study are available from the corresponding author upon reasonable request. Data are stored on secure institutional servers at Mass General Brigham and shared in accordance with institutional policies and participant consent.

## Code availability

Custom code used for data processing and statistical analysis is available from the corresponding author upon reasonable request.
