# Supplementary material for: Bacillus Calmette–Guérin (BCG) immunotherapy reprograms CNS immunity and alters Alzheimer’s biomarkers: results from two open-label clinical trials
Source: Commun Med (Lond). 2026 Jul 2;6:358. doi: 10.1038/s43856-026-01691-7 (PMC13328741; doi:10.1038/s43856-026-01691-7)
Supplement: Supplementary file 1 — Supplementary Information [file 43856_2026_1691_MOESM1_ESM.pdf]

Supplementary Information

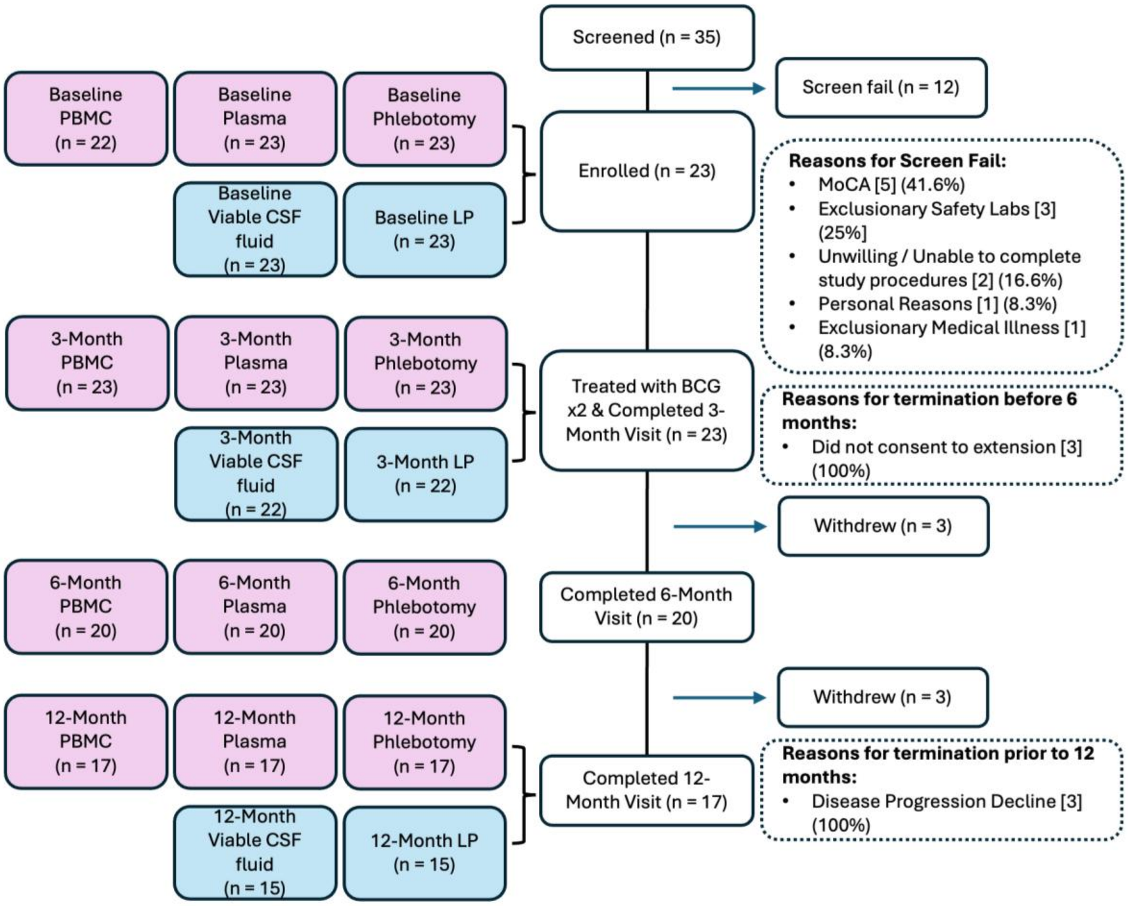

Supplementary Fig 1. Study screening, enrollment and sample collection flow diagram.

A total of 35 individuals were screened across both trials (NCT04507126 and NCT05004688; the latter enrolling participants with confirmed AD pathology), and 23 participants were enrolled and received two doses of BCG. Reasons for screen failure included low MoCA score, safety lab abnormalities, procedural unwillingness, and medical exclusions. All enrolled participants completed the 3-month visit; 3 participants declined further participation following completion of the original 3-month study period prior to protocol extension, resulting in 20 completing the 6-month visit. Three additional participants withdrew due to disease progression, yielding 17 who completed the 12-month visit. PBMC, plasma, CSF, and LP samples were collected at each time point as shown.

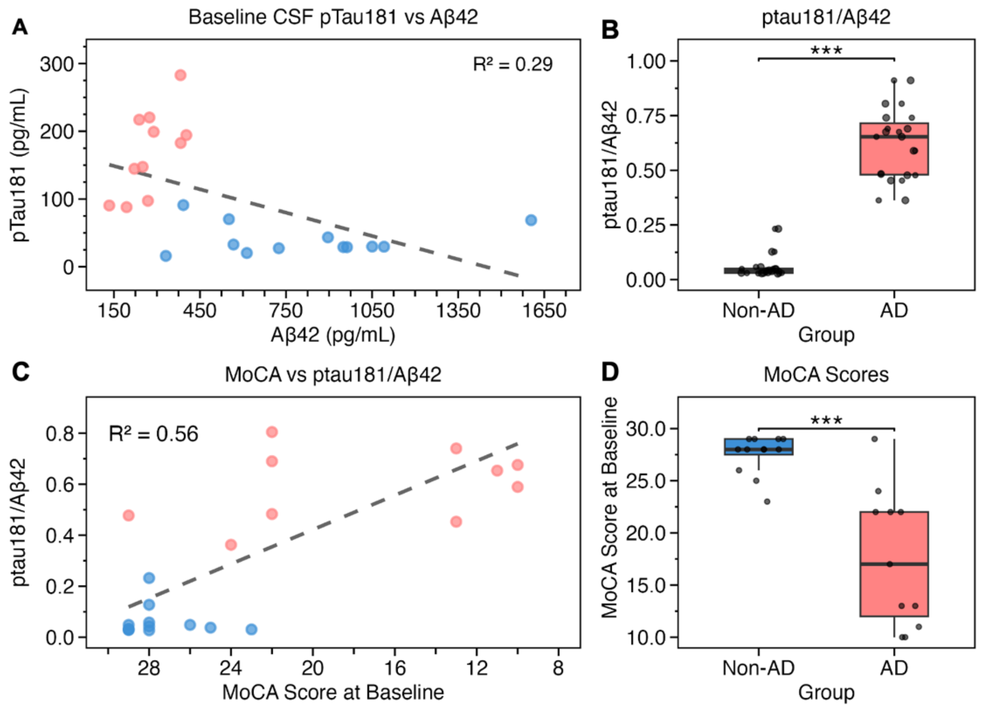

Supplementary Fig 2. Scatterplots and groupwise distributions of CSF biomarkers and cognition, stratified by AD vs non-AD group.

A. Baseline CSF pTau181 and Aβ42 levels are inversely correlated ($R^2 = 0.29$), with group membership (AD vs non-AD) visually separating the data.

B. The pTau181/Aβ42 ratio is higher in the AD group than in non-AD, distinguishing the groups at baseline.

C. MoCA scores show a strong inverse correlation with the pTau181/Aβ42 ratio ($R^2 = 0.56$), with group differences visually separating the distributions.

D. The AD group shows lower MoCA scores than the non-AD group, consistent with greater cognitive impairment.

Triple asterisks (***) indicate $p < 0.001$.

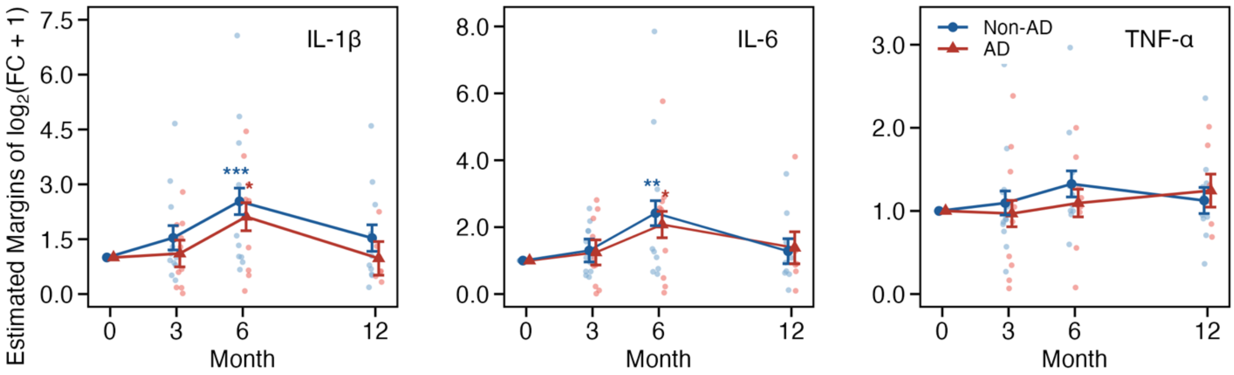

Supplementary Fig 3. Cytokine responses to HKCA stimulation in PBMCs over time following BCG immunotherapy.

HKCA stimulation of peripheral blood mononuclear cells (PBMCs) induced significant increases in IL-1β, IL-6, and TNF-α in both AD and non-AD participants. IL-1β and IL-6 responses peaked at 6 months in both groups, with modestly greater induction observed in non-AD participants. TNF-α levels peaked at 6 months in the non-AD group but did not differ significantly from baseline. In the AD group, TNF-α induction increased between 3 and 12 months, though no timepoint showed a significant difference from baseline. Data represent estimated marginal means of log_2(fold change +1) from linear mixed-effects models with fixed effects for Month, AD status, and their interaction, and subject-level random intercepts. Asterisks indicate within-group significance compared to baseline (*$p < 0.05$, **$p < 0.01$, ***$p < 0.001$); hash marks denote significant AD × Month interactions (#$p < 0.05$, ##$p < 0.01$, ###$p < 0.001$). Error bars represent standard error of the mean (SEM). Statistical tests were two-sided; p values are nominal and not adjusted for multiple comparisons, consistent with the exploratory design. Full model Ns, effect estimates, confidence intervals, and exact p values are reported in Supplementary Data 4.

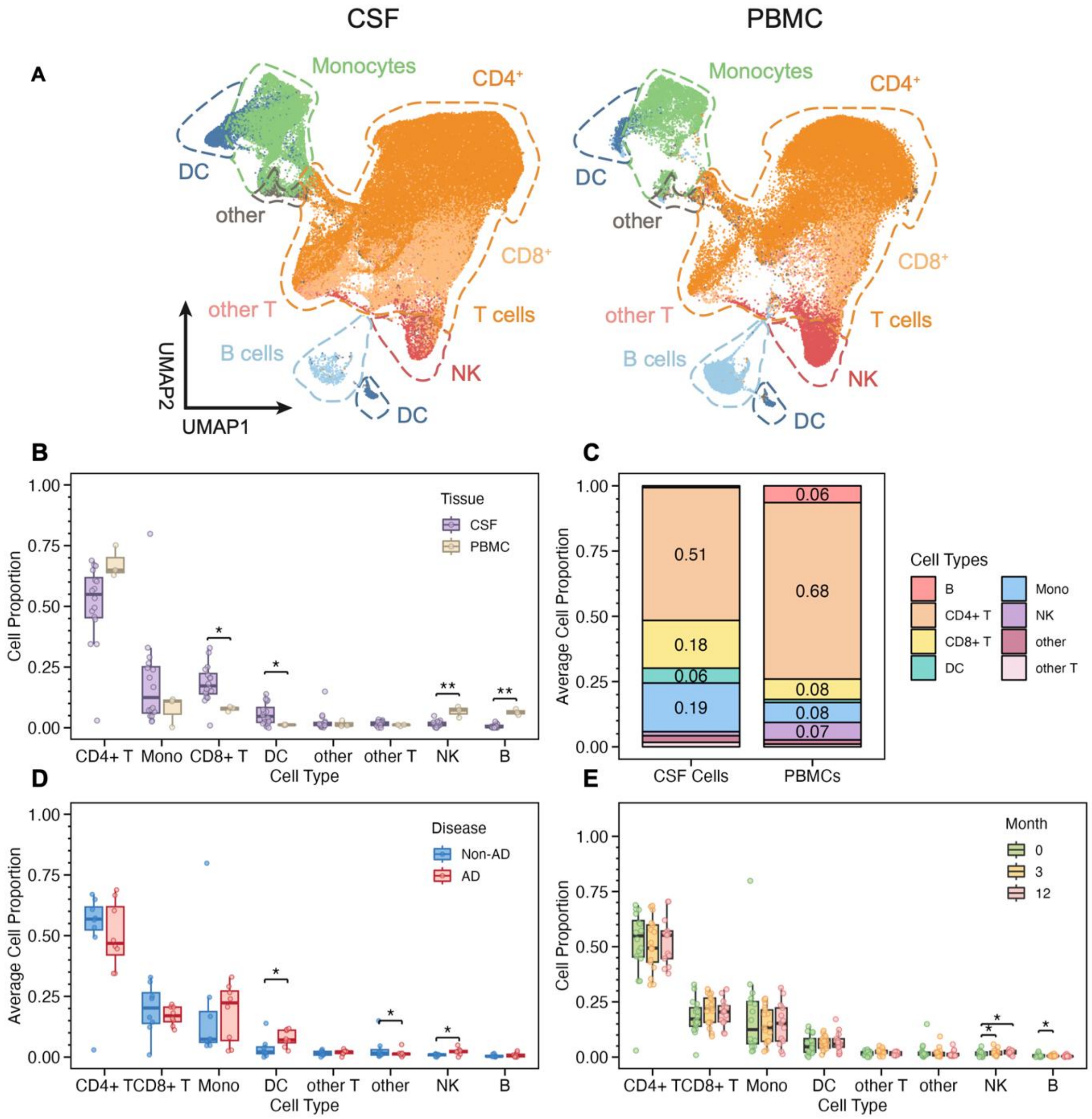

Supplementary Fig 4. Single-cell profiling and immune cell composition in CSF and PBMCs.

(A) UMAP embedding of 254,292 single cells from CSF (n = 106,224) and PBMCs (n = 148,068), annotated using Azimuth reference mapping and integrated with Harmony. Canonical immune lineages include CD4$^+$ and CD8$^+$ T cells, monocytes, NK cells, B cells, dendritic cells (DCs), and other subsets.

(B) Boxplots comparing baseline cell type proportions by compartment. CD8$^+$ T cells and DCs were more abundant in CSF, while NK and B cells were enriched in PBMCs.

(C) Stacked bar plot showing average cell type proportions in CSF and PBMCs.

(D) Boxplots of CSF cell type proportions at baseline by AD vs. non-AD status. NK and DC proportions were modestly higher in AD participants.

(E) Longitudinal CSF cell type proportions at 0, 3, and 12 months post-BCG. No consistent changes were observed across timepoints, though transient increases were seen in B cells at Month 3 and NK cells at Months 3 and 12. Both populations are rare in CSF (B cells: 0.7%; NK cells: 1.6%), and these findings should be interpreted with caution.

Plotted values represent raw cell proportions. Statistical comparisons were performed on logit-transformed proportions using mixed-effects models. Asterisks indicate statistical significant comparisons as annotated (*$p < 0.05$, **$p < 0.01$). Full model outputs are provided in Supplementary Data 4 and 9; raw and transformed values are provided in Supplementary Data 8. Statistical tests were two-sided; p values are nominal and not adjusted for multiple comparisons.

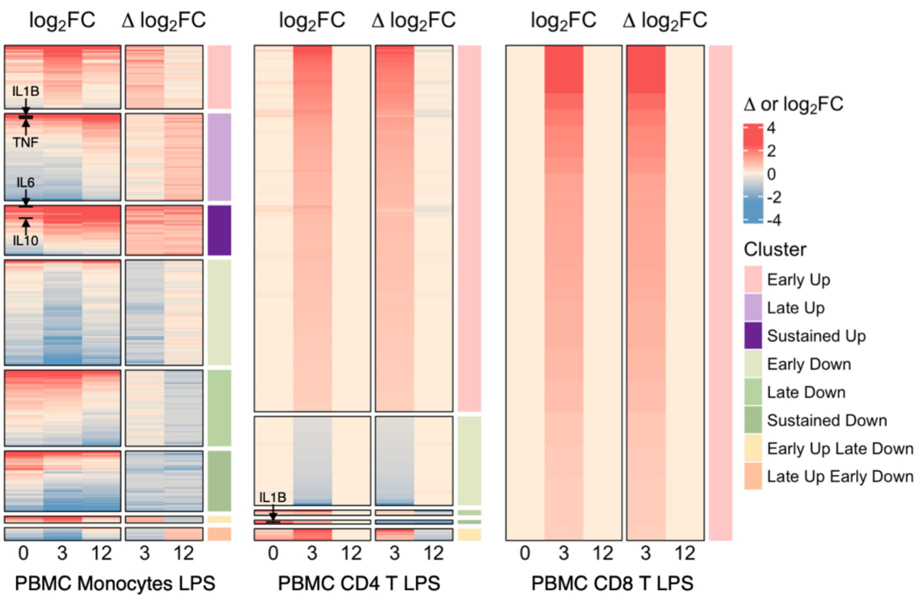

Supplementary Fig 5. Longitudinal transcriptional responses to LPS stimulation in PBMC immune subsets.

Heatmaps show $\log_2$ fold change (FC) in gene expression following LPS stimulation in PBMC monocytes (left), $CD4^+$ T cells (middle), and $CD8^+$ T cells (right), relative to media-only controls at baseline, 3 months, and 12 months (left panels). $\Delta \log_2FC$ represents the change from baseline at post-treatment timepoints (right panels). PBMC monocytes exhibited diverse and robust responses spanning all eight expression clusters, including early, late, and sustained up- and downregulation. IL1B and TNF were enriched in the late up cluster, while IL6 and IL10 were part of the sustained up cluster. $CD4^+$ T cells showed transcriptional responses primarily within the early up cluster. $CD8^+$ T cells also showed upregulation, but responses were restricted to the early timepoint and did not persist at 12 months. These patterns highlight cell type–specific timing and durability of LPS-induced transcriptional reprogramming following BCG immunotherapy.

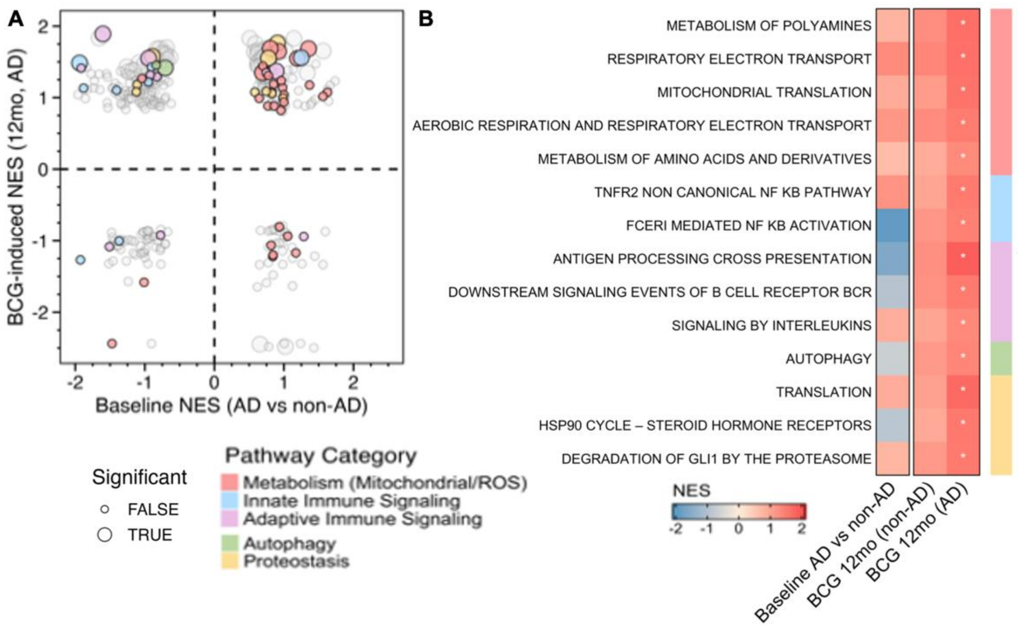

Supplementary Fig 6. Pathway enrichment analysis in CSF CD4⁺ T cells.

(A) Gene set enrichment analysis (GSEA) of curated immune and metabolic pathways in CSF $CD4^+$ T cells. The scatterplot shows normalized enrichment scores (NES) based on baseline AD vs. non-AD (x-axis) and 12-month BCG-induced change within AD (y-axis). Pathways are color-coded by functional category; significant BCG-induced pathways (FDR-adjusted q < 0.05) are shown as larger bubbles. Most significant pathways appeared in the upper right quadrant, indicating pathways enriched in AD at baseline that were further upregulated following BCG.

(B) Heatmap of NES values across three comparisons in $CD4^+$ T cells: baseline AD vs. non-AD, 12-month change in AD, and 12-month change in non-AD. Functional categories match those in (A), and all significant pathways not classified as “other” are shown. Several pathways enriched in AD at baseline—including mitochondrial metabolism, interleukin signaling, and proteostasis—were also modulated following BCG. Directional patterns were generally similar in non-AD participants, consistent with a BCG-driven effect.

All enrichment scores were derived from GSEA using $\log_2$ fold changes as input. Significance was defined as FDR-adjusted, 2-sided q < 0.05. Full pathway-level and gene-level results are available in Supplementary Data 12 and 6. Equivalent analysis for CSF monocytes is shown in Fig 3.

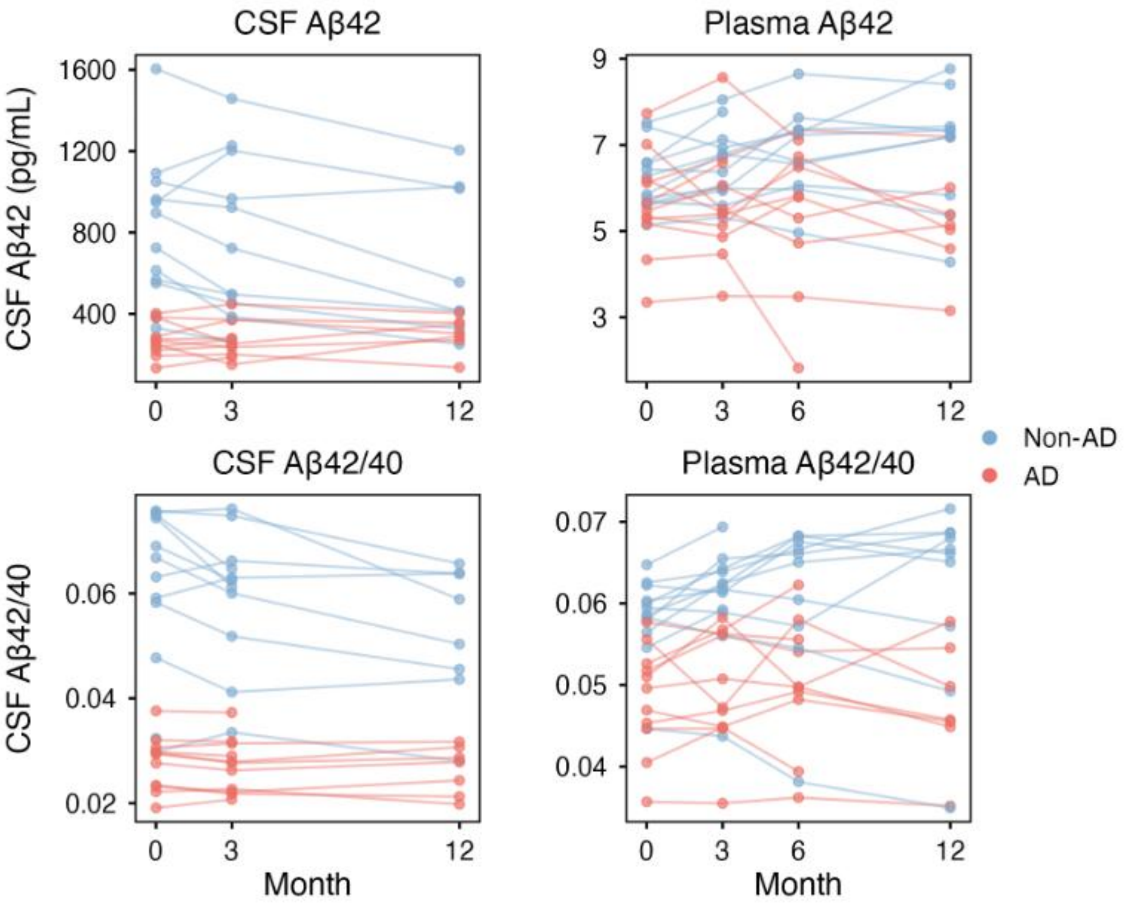

Supplementary Fig 7. Individual trajectories of CSF and plasma amyloid-β measures over time. Individual participant trajectories for CSF Aβ42, CSF Aβ42/40, plasma Aβ42, and plasma Aβ42/40 are shown across study timepoints. Each line represents a single participant. These plots illustrate within-subject variability and complement model-based estimates presented in Fig 5 and Supplementary Data 4. Data are shown pooled across protocols.

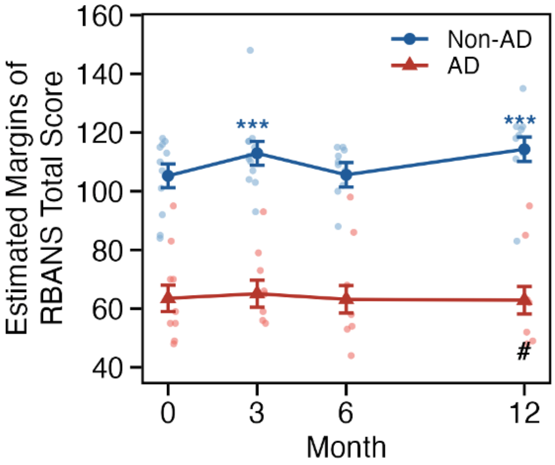

Supplementary Fig 8. Longitudinal RBANS scores by AD group.

Mean RBANS scores for AD and non-AD participants at baseline, 3, 6, and 12 months. Non-AD participants showed modest improvement, likely reflecting test-retest effects, while AD participants remained stable across all timepoints. Data reflect estimated marginal means ± SEM from a linear mixed-effects model. Asterisks indicate within-group comparisons versus baseline (***p < 0.001); hash marks indicate significant AD × Month interaction (#p < 0.05). See Supplementary Data 4 for statistical output. Statistical tests were two-sided; p values are nominal and not adjusted for multiple comparisons, consistent with the exploratory design.
