# Supplementary material for: Bacillus Calmette–Guérin (BCG) immunotherapy reprograms CNS immunity and alters Alzheimer’s biomarkers: results from two open-label clinical trials
Source: Commun Med (Lond). 2026 Jul 2;6:358. doi: 10.1038/s43856-026-01691-7 (PMC13328741; doi:10.1038/s43856-026-01691-7)
Supplement: Supplementary file 2 — Description of Additional Supplementary Files [file 43856_2026_1691_MOESM2_ESM.docx]

Supplementary Data Legends

**Supplementary Data 1. Source data.** Raw biomarker, cytokine, and neuropsychological source data underlying Figures 1, 4, 5 and Supplementary Figures 2, 3, 7, and 8.

**Supplementary Data 2. Participant demographics.** Baseline demographic and clinical characteristics for AD and non-AD groups.

**Supplementary Data 3. Baseline biomarker, immune, and neuropsychological profiles.** Median [IQR], sample size, p-values, and effect sizes for baseline comparisons (raw and log-transformed) across CSF, plasma, PBMC stimulation assays, and neuropsychological measures. Supports Supplementary Figure 2.

**Supplementary Data 4. Longitudinal mixed-effects model outputs.** Full mixed-effects model repeated measures (MMRM) outputs across biomarker, cytokine, immune cell proportion, and neuropsychological endpoints, including model estimates, confidence intervals, exact p-values, and interaction terms; supports Figures 1, 4, 5, and Supplementary Figures 2, 3, 4, 7, and 8.

**Supplementary Data 5. Protocol sensitivity analyses.** Comparison of longitudinal mixed-effects model results with and without inclusion of protocol as a covariate across endpoints, including AD × time interaction coefficients and differences between models.

**Supplementary Data 6. Pathway enrichment and functional categorization.** Reactome pathway enrichment outputs and pathway classification schema used in Figure 3 and Supplementary Figure 6.

**Supplementary Data 7. Summary of adverse events.** Adverse events reported across all enrolled participants, categorized by severity, attribution to study procedures or drug, and seriousness. Events are grouped by system organ class and reported as counts and percentages.

**Supplementary Data 8. Single-cell counts and proportions.** Raw cell counts, calculated proportions, and transformed immune cell proportion data across CSF and PBMC samples by donor, timepoint, and stimulation condition; supports Supplementary Figure 4.

**Supplementary Data 9. Immune cell proportion comparisons.** Nonparametric comparisons of immune cell-type proportions between PBMC and CSF compartments and between AD and non-AD groups, including medians, p-values, and effect sizes; supports Supplementary Figure 4.

**Supplementary Data 10. Differential gene expression following LPS and HKBCG stimulation.** Differentially expressed genes across cell types, timepoints, and compartments, with associated statistical metrics and comparison metadata; supports Figure 2 and Supplementary Figure 5.

**Supplementary Data 11. Longitudinal and pathway analyses of stimulation responses.** Gene expression dynamics and pathway enrichment results across timepoints, including fold-change patterns and functional annotations; supports Figure 2 and Supplementary Figure 5.

**Supplementary Data 12. Basal differential gene expression in CSF cells.** Baseline and longitudinal differential expression analyses under unstimulated conditions across cell types; supports Figure 3 and Supplementary Figure 6.
